# The role of the PI3K/AKT signalling pathway in the corneal epithelium: recent updates

**DOI:** 10.1038/s41419-022-04963-x

**Published:** 2022-05-31

**Authors:** Kuangqi Chen, Yanqing Li, Xuhong Zhang, Rahim Ullah, Jianping Tong, Ye Shen

**Affiliations:** 1 Department of Ophthalmology, the First Affiliated Hospital, School of Medicine, Zhejiang University, Hangzhou, Zhejiang 310003 China; 2 Department of Endocrinology, Children’s Hospital of Zhejiang University School of Medicine, National Clinical Research Center for Child Health, Hangzhou, Zhejiang 310052 China

**Keywords:** Growth factor signalling, Apoptosis, Extracellular matrix

## Abstract

Phosphatidylinositol 3 kinase (PI3K)/AKT (also called protein kinase B, PKB) signalling regulates various cellular processes, such as apoptosis, cell proliferation, the cell cycle, protein synthesis, glucose metabolism, and telomere activity. Corneal epithelial cells (CECs) are the outermost cells of the cornea; they maintain good optical performance and act as a physical and immune barrier. Various growth factors, including epidermal growth factor receptor (EGFR) ligands, insulin-like growth factor 1 (IGF1), neurokinin 1 (NK-1), and insulin activate the PI3K/AKT signalling pathway by binding their receptors and promote antiapoptotic, anti-inflammatory, proliferative, and migratory functions and wound healing in the corneal epithelium (CE). Reactive oxygen species (ROS) regulate apoptosis and inflammation in CECs in a concentration-dependent manner. Extreme environments induce excess ROS accumulation, inhibit PI3K/AKT, and cause apoptosis and inflammation in CECs. However, at low or moderate levels, ROS activate PI3K/AKT signalling, inhibiting apoptosis and stimulating proliferation of healthy CECs. Diabetes-associated hyperglycaemia directly inhibit PI3K/AKT signalling by increasing ROS and endoplasmic reticulum (ER) stress levels or suppressing the expression of growth factors receptors and cause diabetic keratopathy (DK) in CECs. Similarly, hyperosmolarity and ROS accumulation suppress PI3K/AKT signalling in dry eye disease (DED). However, significant overactivation of the PI3K/AKT signalling pathway, which mediates inflammation in CECs, is observed in both infectious and noninfectious keratitis. Overall, upon activation by growth factors and NK-1, PI3K/AKT signalling promotes the proliferation, migration, and anti-apoptosis of CECs, and these processes can be regulated by ROS in a concentration-dependent manner. Moreover, PI3K/AKT signalling pathway is inhibited in CECs from individuals with DK and DED, but is overactivated by keratitis.

## Facts

- CECs are present in the outermost layer of the cornea and play an important role in maintaining CE homeostasis and optical function.
- The PI3K/AKT signalling pathway is widely involved in the proliferation, apoptosis, migration, and other functions of CECs.
- Many corneal diseases and wound healing are associated with the PI3K/AKT signalling and its interaction with ROS.

## Open questions

- How do CECs respond to different environmental conditions, such as health and disease states, through the PI3K/AKT signalling pathway?
- How does the PI3K/AKT signalling pathway interact with ROS in CECs to control the cellular response and influence disease progression?
- Can the PI3K/AKT signalling pathway be used as a major target for the treatment of corneal diseases in the future?

## Introduction

In 1987, Staal first discovered a proto-oncogene, i.e., the *AKT1* gene on human chromosome 14, band q32 [1, 2]. In subsequent years, a lot of studies gradually revealed the intracellular signal transduction cascade centred on phosphatidylinositol 3 kinase (PI3K) and AKT, named the PI3K/AKT signalling pathway. The process of the PI3K/AKT signalling pathway mainly includes binding of exogenous factors to receptors, receptor activation of PI3K phosphorylation, PI3K-induced phosphorylation of AKT, and initiation of downstream effectors [3]. Activated PI3K further catalyses the production of the second messenger phosphatidylinositol-3,4,5-triphosphate (PIP3). The binding of PIP3 to the PH domain and plasma membrane translocation of AKT trigger its phosphorylation [4]. After that, PI3K/AKT activates the phosphorylation or complex formation of downstream molecules, including glycogen synthase kinase 3β (GSK3β), mammalian target of rapamycin (mTOR), and actin-related proteins [5, 6]. These molecules then regulate metabolic functions, such as gluconeogenesis, protein synthesis, cell cycle, migration and apoptosis [7].

**Fig. 1:**
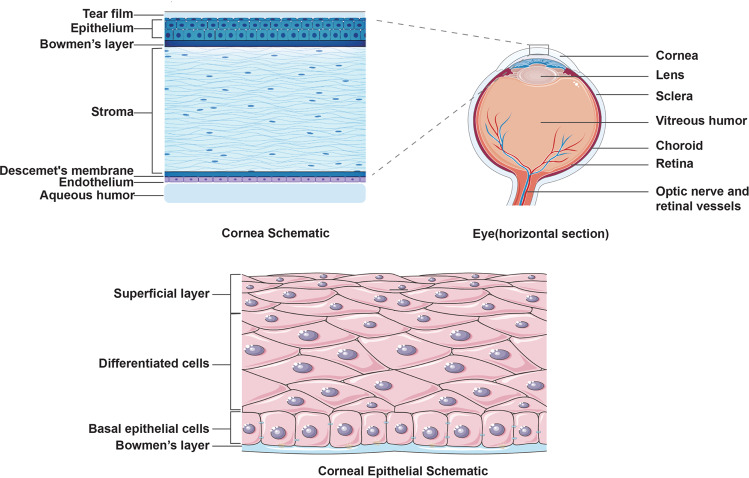
The anatomy of the eye, cornea and corneal epithelium. As the visual organ of the human body, the eye is a complex and sophisticated structure that consists of the cornea, lens, vitreous, choroid, retina, optic nerve, and other accessory structures from front to back. The cornea is situated at the front of the eye and is composed of the CE, Bowman’s layer, corneal stroma, Descemet’s membrane and the endothelium from outside to inside. Located at the outermost layer of the cornea, the CE is derived from the surface ectoderm. The CE is a self-renewing stratified nonkeratinized squamous epithelium that protects the inner eye tissue, forms the immune barrier, supports the tear film, and maintains transparency to play a role in transmitting light. CECs include superficial squamous cells, central suprabasal cells, and a single layer of inner columnar basal cells.

The eye is a complex structure that consists of the cornea, lens, vitreous, choroid, retina, optic nerve, and other accessory structures (Fig. 1). The cornea is composed of the corneal epithelium (CE), Bowman’s layer, corneal stroma, Descemet’s membrane and the endothelium. The CE is a self-renewing stratified nonkeratinized squamous epithelium that protects the inner eye tissue, forms the immune barrier, supports the tear film, and maintains transparency [8, 9]. Corneal epithelial cells (CECs) include superficial squamous cells, central suprabasal cells, and a single layer of inner columnar basal cells [10]. During CE homeostasis, the adult CE is maintained by a comprehensive process involving cell proliferation, migration, differentiation and apoptosis. CECs undergo inflammation, apoptosis and/or autophagy when the CE is damaged, and the adjacent basal cells of the CE secrete a large number of cytokines, growth factors to promote proliferation, differentiation and migration, ultimately achieving wound healing by regulating signalling networks in CECs [11].

Diabetic keratopathy (DK) is caused by diabetes-associated hyperglycaemia (a persistent high-glucose state in blood in general) and manifests as persistent corneal epithelial erosion, superficial punctate keratopathy, delayed epithelial regeneration, and reduced corneal sensitivity, which may cause impaired vision or permanent vision loss [12]. Dry eye disease (DED) is a multifactorial and chronic disease characterized by tear deficiency and/or evaporative dry eye, and causes the tear film covering the CE to adopt a local hyperosmotic state, which causes damage to the CE and delayed wound healing [13]. Keratitis is a general term describing inflammatory damage to the cornea resulting from various causes, such as microbial infection, neurotrophin deficiency, trauma, and autoimmune disorders. Infectious keratitis refers to keratitis caused by microbial infection, and keratitis from other causes is called noninfectious keratitis. Corneal epithelial defects and delayed wound healing caused by inflammation are important clinical manifestations of keratitis [14, 15].

The PI3K/AKT signalling pathway plays an important role in the biological responses of CECs [16–19]. Importantly, dysregulation of CE homeostasis caused by impaired CEC responses is associated with many corneal diseases, such as DK, DED, and keratitis [18, 20, 21]. Therefore, an in-depth understanding of the pathophysiology of CE with a focus on the PI3K/AKT signalling pathway is important for maintaining the integrity of patients’ corneas and seeking reliable treatments and new drugs [22, 23]. In this review, we provide evidence for the regulatory role of the PI3K/AKT signalling pathway in CECs to support its clinical application.

## Methods

We searched the PubMed database (https://pubmed.ncbi.nlm.nih.gov/) using the keywords “AKT AND corneal epithelial cells” and “AKT AND corneal epithelium”. Afterwards, we carefully read and categorized the results, removed some irrelevant articles, and then focused on recent studies and reviews (published between 2015 and 2022). Additional studies were discovered by consulting the reference lists of the selected articles. Most of the references cited in this review are experimental articles, although a few review articles, case reports, and book chapters are included; no other types of literature are cited. Our figures were edited with Adobe Illustrator CC 2018 software (Adobe, San Jose, CA).

## Roles of growth factors and other hormones in the regulation of CEC functions

**Fig. 2:**
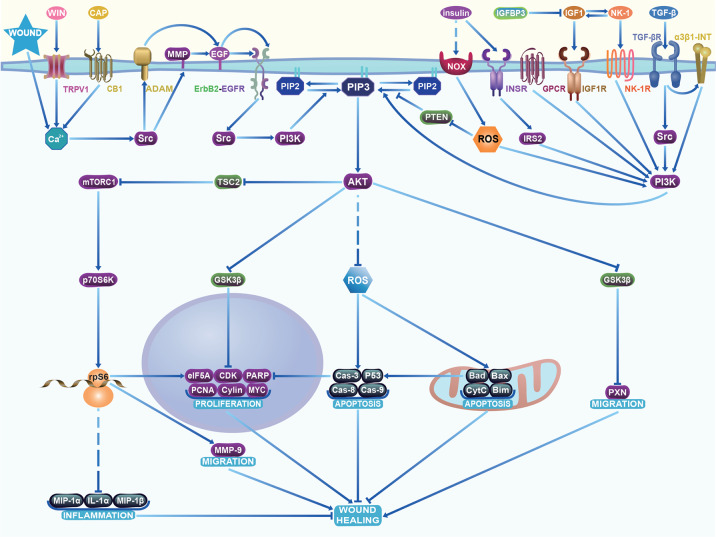
The PI3K/AKT signalling pathway in CECs. Damage to CECs, WIN, and CAP can increase the Ca^2+^ concentration in the cytosol, respectively, which induce ectodomain shedding of EGF (and/or HB-EGF) via activation of ADAM and/or MMPs in the plasma membrane and extracellular matrix. The growth factors, insulin, and NK-1 induce PI3K phosphorylation by binding to their receptors in the plasma membrane. Activated PI3K subsequently phosphorylates lipids on the cell membrane to form the second messenger PIP3, leading to the phosphorylation of AKT. Then, AKT mediates downstream responses by phosphorylating or inducing the formation of complexes composed of various downstream molecules, such as mTOR, ROS, and GSK3β. Specifically, mTOR is an important molecule downstream of AKT, and AKT activation indirectly activates mTORC1, promoting cell migration and proliferation, and inhibiting inflammation and apoptosis. AKT, an antiapoptotic factor, also inhibits cell apoptosis through eliminating excessive ROS in cytosol. However, produced by NADPH oxidase, a low or moderate level of ROS act as a second messenger of PI3K and inhibitor of Phosphatase and tensin homologue, both of which promote activation of AKT. Activated AKT also phosphorylates GSK3β and reduces its activity, thereby promoting cell migration and proliferation. All of the above effects promote the healing of the CE together. NOX, NADPH oxidase; INSR, Insulin receptor; IGFBP3, Insulin-like growth factor-binding protein 3; α3β1-INT, α3β1 integrin; IRS2, Insulin receptor substrate 2; TSC2, Tuberous sclerosis complex 2; Bax, Bcl-2-associated X protein; Bad, Bcl-2-associated agonist of cell death protein; Bim, Bcl-2-like protein 11; CytC, cytochrome c; PXN, Paxillin; PCNA, Proliferating cell nuclear antigen; CDK, Cyclin-dependent kinase; PTEN, Phosphatase and tensin homologue; WIN, MAP kinase kinase kinase win; CAP, capsaicin; CB1, cannabinoid receptor 1; TRPV1, transient receptor potential vanilloid 1; PARP, poly (ADP-ribose) polymerase; MIP-1β, macrophage inflammatory protein 1β; MIP-1α, macrophage inflammatory protein 1α; ADAM, a disintegrin and metalloproteinase.

Growth factors and hormones activate PI3K/AKT signalling in CECs, which regulates apoptosis, proliferation, migration, and wound healing (Table 1, Fig. 2).

**Table 1:** Classic molecules in PI3K/AKT signalling in CECs.

Molecular name	Molecular function	Biological Function in CECs	Regulators	Subcellular location	Contributors
Phosphatase and tensin homologue	Tumor sup ressor, inhibit PI3K/AKT signalling	Apoptosis and inhibition of proliferation	/	Cytoplasm, cell cortex	[66]
PIP2, PIP3, PI3K	Direct regulators of AKT	Proliferation and anti-apoptosis	Phosphatase and tensin homologue	Cytoplasm, cell cortex	[66]
AKT	Upstream regulator of mTORC1, Forkhead box protein O1, and GSK3α/GSK3β	Proliferation and anti-apoptosis	PIP3, PI3K	Cytoplasm, nucleus	[66]
Tuberous sclerosis complex 2, mTOR complex 1 (mTORC1)	Substrates of AKT	Growth and anti-apoptosis	AKT	Cytoplasm	[43]
Forkhead box protein O1	Inhibited by AKT	Apoptosis and inhibition of proliferation	AKT	Cytoplasm and nucleus	[66]
Cyclic AMP-responsive element-binding protein 1	Transcription of Bcl-2	Proliferation and anti-apoptosis	AKT	Nucleus, Golgi apparatus, endoplasmic reticulum membrane	[95]
GSK3β	Activated by AKT, inhibiting GYS1/2 to reduce Glycogen synthesis, inhibiting MYC	Apoptosis and anti-proliferation	AKT	Cytoplasm	[49]
p70S6K, rpS6	Substrate of AKT1/mTORC1, related to Glucose homeostasis and protein synthesis	Proliferation and anti-apoptosis	mTORC1	Cytoplasm	[115]
eIF5A, Eukaryotic translation initiation factor 4E-binding protein 1	Target of p70S6K	Proliferation and anti-apoptosis	p70S6K, rpS6	Nucleus and cytosol	[25]
Cyclin A1/2 Cyclin D1/3 Cyclin E1/3	Regulating the G1/S/G2/M transitions	Proliferation	S-phase kinase-associated protein 2, Myc proto-oncogene protein	Nucleus	[41]
Proliferating cell nuclear antigen	DNA damage response, DNA repair	Proliferation and anti-apoptosis	eIF5A	Nucleus	[116]
Bcl-2	Activated by Cyclic AMP-responsive element-binding protein 1, inhibiting apoptosis	Proliferation and anti-apoptosis	Cyclic AMP-responsive element-binding protein 1	Cytoplasm	[63]
Bcl-2-associated X protein, Bcl-2-like protein 11, Bcl-2-associated agonist of cell death protein, Cytochrome c	Apoptosis regulator in mitochondria	Mitochondria-induced apoptosis	ROS	Mitochondrion	[63, 98]
Cas-3, 8, 9	Apoptosis caused by DNA damage and cleaving poly (ADP-ribose) polymerase	Apoptosis	Cytochrome c	Cytoplasm	[115]
P53	Tumor suppressor, stimulating Bax, inhibiting Bcl-2	Apoptosis	mTORC1	Nucleus and mitochondrion	[40, 76]
A disintegrin and metalloproteinase 9, 10, 12, 17 MMP-3, 8, 9, 10	Local proteolysis of extracellular matrix (fibronectin, gelatins, collagens), establishing and maintaining gradients	Migration	rpS6, Src, Wound	Extracellular matrix, plasma membrane	[117, 118]
IL-1β, IL-1α	Potent proinflammatory cytokine	Inflammation	LPS, ROS, TNF-α	Lysosome, cytosol	[48]
TNF-α	Pathogen defense, inflammation	Inflammation and apoptosis	/	Plasma membrane	[96]

### Epidermal growth factor receptor (EGFR) ligands

Through matrix metalloproteinase (MMP)-dependent ectodomain shedding and transactivation of EGFR signalling (proto-oncogene tyrosine-protein kinase Src/PI3K/AKT), EGFR ligands, including epidermal growth factor (EGF), transforming growth factor-α, and heparin-binding EGF-like growth factor (HB-EGF), inhibit apoptosis and promote proliferation, migration in CECs, and healing of the CE [24].

Eukaryotic translation initiation factor 5A (eIF5A), a nucleocytoplasmic shuttle protein, is the substrate of the EGF-induced EGFR/Src/PI3K/AKT/mTOR/p70S6K/rpS6 signalling pathway and induces the expression of MMP-9 in the extracellular matrix and proliferating cell nuclear antigen in vitro [25, 26]. Furthermore, in mouse CECs in vivo, excess transforming growth factor-α expression promotes the expression of proliferating cell nuclear antigen likely by transactivating the EGFR/PI3K/AKT cascade. MMP-9 mediates the ectodomain shedding of EGF and/or HB-EGF, further activating EGFR/Src/PI3K/AKT signalling by increasing local proteolysis. Proliferating cell nuclear antigen facilitates proliferation by inducing DNA duplication through an increase the activity of DNA polymerase and eIF5A [25, 26].

The HB-EGF/EGFR/Src cascade is a target of various endogenous and exogenous molecules. Furthermore, the influx of extracellular Ca^2+^ increases the activity of Src, and a disintegrin and metalloproteinase and transactivating the HB-EGF/EGFR/Src/PI3K/AKT cascade [27, 28]. In addition, endogenous anti-inflammatory mediators, resolvins, induce MMP-mediated activation of the HB-EGF/EGFR/PI3K/AKT/GSK3β cascade, which inactivates paxillin to inhibit the activation of its substrate focal adhesion kinase and induce the formation of the mitogen-activated protein kinase 3/1-focal adhesion kinase-paxillin complex [29, 30]. These effects increase the migration of human CECs (hCECs) and healing of the CE in vitro [29, 30].

The endocannabinoid system regulates the proliferation and migration of CECs partially by transactivating or inhibiting the EGFR/PI3K/AKT signalling pathway [31, 32]. Two endogenous metabolites, capsaicin and MAP kinase kinase kinase win, and their receptors, cannabinoid receptor 1 and transient receptor potential vanilloid 1, respectively, were identified in previous studies. Activated cannabinoid receptor 1 and transient receptor potential vanilloid 1 increase the migration and proliferation partially through Ca^2+^ influx-induced transactivation of EGFR/Src/PI3K/AKT signalling in hCECs and mouse CECs in vitro [32, 33]. However, in bovine CECs in vitro, endogenous activation of cannabinoid receptor 1 antagonizes the transactivation of the EGFR/PI3K/AKT signalling pathway and promotes migration by inducing chemotaxis rather than inducing proliferation [34]. This opposite outcome may be attributed to the differences among humans, mice, and bovines, and we propose that related experiments on the corneas of other species are needed to further explore the effect of the cannabinoid system on the EGFR/PI3K/AKT cascade in CECs. More importantly, further, in vivo experiments on the endocannabinoid system-EGFR/PI3K/AKT cascade are also necessary.

### Insulin-like growth factor 1 (IGF1) and insulin

In hCECs, IGF1 binds to the IGF1 receptor to activate the insulin receptor substrate 2/PI3K/AKT signalling pathway and activate proliferation, but these effects are inhibited by IGF-binding protein 3 in CECs [35–38]. Furthermore, GSK3β may be phosphorylated by the insulin/insulin receptor /insulin receptor substrate 2/PI3K/AKT signalling cascade to induce degradation of the insulin receptor voltage-dependent anion channel-1 complex at the mitochondrial membrane, leading to senescence, mitophagy, and CEC survival in vitro [39].

Other growth factors, including transforming growth factor-β (TGF-β), hepatocyte growth factor (HGF), keratinocyte growth factor, nerve growth factor, vascular endothelial growth factor B, and pigment epithelium-derived factor, also activate the PI3K/AKT signalling, which promotes the proliferation, migration of CECs, inhibits CEC apoptosis and promotes healing of the CE [40–44].

The relationship between growth factors and DK is discussed in section 5.2.

### Neurokinin 1 (NK-1)

As a neuropeptide that is widely distributed in nerve fibres, NK-1 (also called substance P) facilitate healing in the CE and the recovery of mitochondrial function in CECs [45, 46]. By binding to the NK-1 receptor, NK-1 restores EGFR/AKT/GSK3β signalling, which eliminates Caspase-3 (Cas-3) and reactive oxygen species (ROS) to inhibit CEC apoptosis under hyperglycaemic and hyperosmolar conditions [45, 47–50]. Cas-3 with reduced expression and activity cannot execute apoptosis in cytosol [50]. In addition, activation of AKT signalling partially inhibits the production of interleukin (IL)-1α, macrophage inflammatory protein 1α, and macrophage inflammatory protein 1β, all of which are associated with local inflammation and delayed healing of the CE [48]. Moreover, the NK-1 and C domains of IGFs synergistically activate the PI3K/AKT/GSK3β signalling pathway to promote cell migration [38, 51, 52].

## Interactions between ROS and AKT in CECs

ROS are the byproducts of oxygen and are produced due to cellular metabolism, and regulate the redox balance, proliferation, cytotoxicity, and metabolic adaptation in a concentration-dependent manner [47, 53–56]. In the CE, ROS production is triggered by damage outside the eye (including chemical- or UV light-induced damage, and mechanical damage), as well as by inflammatory factors in the aqueous humour or tears [57–59].

### Extreme environments-induced excessive accumulation of ROS

Extreme environments including hyperglycaemic and hyperosmolar environments may cause excess ROS accumulation, which is considered an upstream suppressor of the EGFR-mediated PI3K/AKT signalling pathway, and delays healing of the CE [60]. In vitro and in vivo studies have shown that inhibition of ROS production reactivates PI3K/AKT signalling, which decreases apoptosis and inflammation in CECs [22, 23]. Furthermore, the expression of the cellular tumour antigen P53, a PI3K/AKT substrate, is upregulated by ROS in hCECs [61, 62]. In this manner, ROS increase Bcl-2, Bcl-2-associated X, and cytochrome c levels in mitochondria, and Cas-3, Cas-8, and Cas-9 levels in the cytosol and cleave poly (ADP-ribose) polymerase in the nucleus. These proteins are all substrates of PI3K/AKT signalling and apoptosis-related proteins [61, 63].

The role of ROS in diabetes and hyperglycaemia is discussed in section 5.1.1.

### Low or moderate levels of ROS

Interestingly, ROS production may also be induced by growth factors, and may function as a second messenger to activate PI3K/AKT signalling at low or moderate levels. These effects stimulate the proliferation and migration of healthy CECs, inhibit apoptosis of healthy CECs, and induce healing of the CE in vitro (in human and rabbit CECs) and ex vivo (in pig cornea) [64, 65]. Although no studies have investigated the targets of the AKT signalling pathway in CECs, a study in another cell type indicated that the possible targets of ROS in the PI3K/AKT signalling pathway include phosphatase and tensin homologue, the P85 domain of PI3K [66]. Phosphatase and tensin homologue impairs PI3K/AKT signalling via the hydrolysis of phosphorylation sites of PI3K, PIP2, and PIP3, resulting in delayed proliferation and migration of CECs [67]. Studies in CECs and lens epithelial cells suggest that the production of very low levels of ROS may be triggered by growth factors and produced by NADPH oxidase in the plasma membrane rather than mitochondria to activate PI3K/AKT signalling, however, this result requires further confirmation in CECs [64, 68–70].

## Diseases, their therapies, and AKT in CECs

**Fig. 3:**
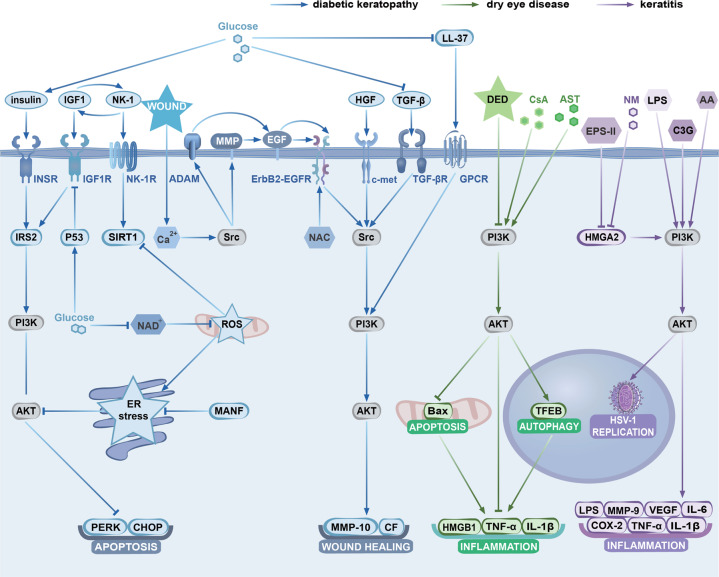
The roles of different molecules related to the PI3K/AKT pathway in CECs in diseases and therapeutic strategies. The PI3K/AKT signalling pathway plays an important role in DK, DED and keratitis. Many exogenous or endogenous molecules affect the development of corneal diseases through the PI3K/AKT signalling pathway. Furthermore, many newly discovered drugs alleviate these corneal diseases by regulating the PI3K/AKT signalling pathway. This figure uses different colours to indicate different corneal diseases and clarify the complex signalling network in CECs. In DK, hyperglycaemia induces insulin production, promotes the activation of the PI3K/AKT signalling pathway, and inhibits cell apoptosis. On the other hand, hyperglycaemia increases ROS levels. It inhibits IGF1 receptor, EGFR-ErbB2, TGF-β, GPCR, and other AKT upstream molecules, inducing cell apoptosis and inhibiting corneal wound healing. DED-induced hyperosmolarity inhibits the PI3K/AKT pathway and activates cell apoptosis and autophagy, leading to corneal inflammation. CsA and AST were also discovered reverse the changes caused by DED through the PI3K/AKT pathway. However, in noninfectious and infectious keratitis, overactivated AKT promotes inflammation. LPS, NM, and EPS-II increase the levels of inflammation-related proteins by activating the PI3K/AKT pathway. Moreover, as potential drugs, AA, C3G, and other molecules inhibit inflammation through the PI3K/AKT pathway. In particular, in viral keratitis, the PI3K/AKT pathway is also an important target for regulating viral (HSV-1) replication. GPCR, G protein-coupled receptor; AA, asiatic acid; NM, Nitrogen mustard; NAC, N-acetyl cysteine; MANF, Mesencephalic astrocyte-derived neurotrophic factor; TFEB, Transcription factor; PERK, Protein kinase RNA- like endoplasmic reticulum kinase; CHOP, C/EBP-homologous protein; VEGF, Vascular endothelial growth factor; COX2, Cyclooxygenase 2; CF, cathepsin F; HSV-1, herpes simplex virus type 1.

In the healthy CE, the levels of phosphorylated PI3K and AKT are relatively low to maintain the homeostasis of CECs. When the CE is damaged, growth factors and NK-1 are secreted to activate the PI3K/AKT cascade, which promotes proliferation and migration and inhibits apoptosis and inflammation of CECs. These effects together enable the healing of the CE. However, under the influence of various factors (such as hyperglycaemia, hyperosmolarity, excess inflammatory factors and cytokines, toxicants, and microbial infection), both the CE homeostasis and the normal expression of the PI3K/AKT signalling pathway are disrupted. These processes are associated with many diseases, including DK, DED, and keratitis (Table 2, Fig. 3). Hence, molecules targeting the PI3K/AKT signalling pathway have become promising therapies [22, 71–73].

**Table 2:** The function of different molecules related to PI3K/AKT pathway in CECs and CECs-related diseases.

Molecular name	Molecular function	Biological function in CECs	Regulators	Subcellular location	Contributors
HB-EGF	EGFR and ErbB2 binding	Wound healing, cell migration, proliferation	Src; MMP 3, 7;a disintegrin and metalloproteinase 9, 10, 12, 17	Plasma membrane	[116, 119]
EGF, EGFR	Activating PI3K/AKT signalling	Anti-apoptosis	HB-EGF	Plasma membrane	[119]
Poly (ADP-ribose) polymerase	DNA repair	Anti-apoptosis	Cas-3, 8, 9	Nucleus	[120]
ErbB2	Receptor of HB-EGF	Anti-apoptosis	HB-EGF	Plasma membrane and nucleus	[121]
Src	Inducing ectodomain shedding of HB-EGF, and playing as second messenger of EGFR	Proliferation and anti-apoptosis	Ca^2+^, EGFR, ErbB2	Cytoplasm	[120]
TGF-β	Activate PI3K/AKT and MAPK signalling pathway	Epithelial mesenchymal transition, keratitis, wound healing	Peucine-rich alpha-2-glycoprotein	Extracellular region	[122]
MAPK(P38)	Inhibtied by EGF signalling and inhibiting TGF-β	Apoptosis	EGF	Cytosol and nucleus	[123]
Transforming Growth Factor β-Induced Protein	Induced by TGF-β, binding with α3β1 integrin	Adhesion	TGF-β	Extracellular matrix	[124]
eIF5A, PCNA	The substrate of EGF/EGFR/Src/PI3K/AKT signalling pathway	Cell migration and proliferation	EGF	Nucleus	[25]
MMP-9	The substrate of EGF/EGFR/Src/PI3K/AKT signalling pathway	Cell migration and proliferation	EGF, NM	Cytosol and extracellular matrix	[25]
LPA	Transactivate EGFR and activate Src/PI3K/AKT signalling pathway	Wound healing, inflammation	Protein phosphatase 2	Extracellular region	[113, 114]
G protein coupled receptor	transactivation of AKT signalling	Proliferation and anti-apoptosis	LPA	Plasma membrane	[125]
P2Y purinoceptors	Multi-pass membrane protein for influx of extracellular Ca^2+^	Proliferation and anti-apoptosis	ATPγS	Plasma membrane	[126]
LL-37	Bind to LPS and transactivate EGFR	Proliferation, anti-apoptosis and anti-bacterial	Wound	Plasma membrane	[126]
Capsaicin, cannabinoid receptor 1	Mobilization from intracellular Ca^2+^ stores	Proliferation and anti-apoptosis	/	Plasma membrane and nucleus	[33]
Mitogen-activated protein kinase kinase kinase win, transient receptor potential vanilloid 1	Influx of extracellular Ca^2+^	Proliferation and anti-apoptosis	/	Plasma membrane and nucleus	[33]
Paxillin	Cytoskeletal protein associated with actin-membrane attachment, correlating with integrin	Adhesion(anti-migration)	GSK3β	Cell cortex and cytoskeleton	[127]
Focal adhesion kinase	Activation of PI3K/AKT, maintaining focal adhesion, binding with cytosol part of integrin	Adhesion(anti-migration), proliferation, anti-apoptosis	Paxillin, Integrin	Nucleus, plasma membrane, cytoskeleton	[127]
IGF1, IGF1R	Activating PI3K/AKT signalling	Cell adhesion, proliferation, survival	Insulin, IGF binding protein 3	Nucleus, plasma membrane, cytoplasm	[35, 76]
IGFBP3	Binding with IGF1 to inhibit IGF1/IGF1R	Apoptosis, adhesion	P53	Secreted	[128]
Leucine-rich alpha-2-glycoprotein 1	Activating TGFβR, promoting the expression of MMP-3 and MMP-13	Wound healing	Glucose	Secreted	[122]
SIRT1	Enhancing IGFBP3/IGF-1R/AKT pathway	Proliferation and anti-apoptosis	NK-1R, p53	Nucleus, cytoplasm, mitochondrion	[76]
Insulin, INSR	Activating PI3K/AKT signalling, blocks GSK3β activity	Wound healing, cell migration, mitophagy and mitochondrial accumulation	Glucose	Extracellular region	[39, 129]
HGF, Keratinocyte growth factor	Activating PI3K/AKT/p70S6K signalling pathway	Wound healing and cell proliferation	IL-1, IL-6, TNFα, glucose	Extracellular region	[40]
Nerve growth factor	Activating PI3K/AKT signalling pathway, reduce the expression of Caspase-3,9, Bad, Bax and Bim	Cell growth and G1-S transition	/	Endosome and extracellular region	[41]
Vascular endothelial growth factor-B, Vascular endothelial growth factor receptor-1	Promote the expression of pigment epithelium-derived factor via PI3K/AKT/GSK3β/mTOR signalling	Wound healing and regeneration of nerve fiber	Vascular endothelial growth factor trap	Extracellular region	[43]
NK-1, NK-1R	Reactivate EGFR, AKT, and SIRT1 signalling	Wound healing, corneal sensation recovery and mitochondrial function recovery	NK-1 receptor antagonist	Extracellular region and plasma membrane	[45]
ROS	Suppressor of EGFR-mediated PI3K/AKT signalling pathway, increase the expression of Bcl-2, Bax, cytochrome c, Caspase-3, 8, and 9	Delay wound healing, delay regeneration and migration, apoptosis, Inflammation, keratoconus	N-acetylcystein	Cytoplasm and mitochondrion	[53–55, 61]
MAPK3/1	MAPK3/1 signalling pathway synergistics with PI3K/AKT signalling pathway	Anti-apoptosis, wound healing, cell survival	Dual specificity mitogen-activated protein kinase kinase mek	Cytoplasm and cytoskeleton	[130]
NAD^+^	Increasing ROS, inhibit SIRT1/EGFR/PI3K/AKT signalling pathway	Mitochondria-induced apoptosis, delay wound healing in diabetes	Glucose	Cytoplasm and mitochondrion	[77]
Nicotinamide phosphoribosyltransferase	Increasing ROS, inhibit SIRT1/EGFR/PI3K/AKT signalling pathway	Mitochondria-induced apoptosis, delay wound healing in diabetes	Glucose	Cytoplasm, nucleus, extracellular region	[77]
Mesencephalic astrocyte-derived neurotrophic factor	Activate AKT signalling	Inhibits hyperglycaemia-induced ER stress and ER stress-mediated apoptosis, wound healing, nerve regeneration in diabetes	Glucose	Endoplasmic reticulum and extracellular region	[85]
MMP-10, Cathepsin F	Downstream of EGFR/PI3K/AKT signalling pathway	Delay wound healing in diabetes	Glucose	Extracellular region and lysosome	[89]
Ephrin-A1, A2	Suppressing AKT signalling	Attenuates cell migration, delay wound healing in diabetes	Glucose	Plasma membrane and extracellular region	[131]
HMGB1	Related to PI3K/AKT signalling	Immune response, tissue damage in DED	AST	Plasma membrane, nucleus, endosome cytoplasm, extracellular region	[96]
CsA	Inhibit the expression of TNF-α, Bax and Bcl-2 via reactivating PI3K/AKT signalling pathway	Reduce apoptosis and inflammation	/	Extracellular region and cytoplasm	[98]

### Diabetes-associated hyperglycaemia inhibits PI3K/AKT signalling to induce apoptosis and inflammation, and delay wound healing

Diabetes-associated hyperglycaemia stimulates apoptosis and inflammation but inhibits migration and tight junctions in CECs, thus causing DK [20].

#### Hyperglycaemia-induced excess ROS accumulation inhibits PI3K/AKT signalling to increase apoptosis and delay wound healing

Hyperglycaemic conditions induce mitochondrial fragmentation, which leads to ROS overproduction that inactivates PI3K/AKT [74]. Silent information regulator 1 (SIRT1) is a substrate of PI3K/AKT signalling that decreases ROS production, apoptosis, and endoplasmic reticulum (ER) stress in CECs in vitro [75]. Hyperglycaemia reduces SIRT1 expression, increases P53 acetylation, and inhibits the IGF1/PI3K/AKT cascade in CECs, which may cause delayed healing of the CE in vivo and in vitro [76, 77].

Antioxidant therapy, which induces the clearance of excess ROS and activates the AKT pathway, is an effective drug treatment strategy for DK. For example, N-acetylcysteine reactivates the PI3K/AKT signalling pathway by inhibiting excess ROS production and restoring CEC migration and healing of the diabetic human and pig CE in vitro and ex vivo [60]. Similarly, exogenous NAD^+^ and its precursors nicotinamide mononucleotide and nicotinamide riboside attenuate ROS accumulation, restore the mitochondrial membrane potential and facilitate the healing of the CE and regeneration of corneal nerves [77]. Moreover, NK-1 repairs mitochondrial damage and clears accumulated ROS, thus improving diabetic CE wound healing and corneal sensation [45, 78, 79].

#### Hyperglycaemia-induced ER stress suppresses AKT to inhibit wound healing

ER stress inhibits the PI3K/AKT signalling pathway and causes apoptosis and inflammation [80]. In individuals with DK, an imbalance in ROS levels causes ER stress [81, 82]. A study examining diabetic rabbit CECs found that hyperglycaemia induces the expression of protein kinase RNA-like ER kinase and C/EBP-homologous protein, inducing ER stress and subsequently inhibiting the AKT signalling pathway in activate apoptosis in vitro [74].

Interestingly, inhibition of ER stress activates the AKT pathway and promotes healing of the CE. As neuroprotective factors, most mesencephalic astrocyte-derived neurotrophic factors are retained in the ER and play an important role in maintaining ER stability [83, 84]. Moreover, mesencephalic astrocyte-derived neurotrophic factors inhibit hyperglycaemia-induced ER stress, which promotes wound healing and nerve regeneration in the CE under normal and diabetic conditions through AKT activation [85].

#### Hyperglycaemia-induced inactivation of growth factor receptors inhibits PI3K/AKT signalling, increases apoptosis, and delays wound healing

Hyperglycaemia inhibits the expression of growth factor receptors in CECs. In the context of diabetes, the expression of the c-met (also called HGF receptor), which may directly inhibit HGF/c-met/PI3K/AKT signalling, is decreased in CECs in vitro [86]. The expression and phosphorylation/activity of c-met are restored by recombinant adenovirus-driven c-met overexpression, which normalizes wound healing and the expression of diabetes markers in diabetic hCECs [86]. This effect may be explained by the interactions between the HGF/c-met system and other growth factors including IGF and EGF [87, 88].

Inactivation of the EGFR/PI3K/AKT signalling pathway leads to the overexpression of MMP-10 and cathepsin F. MMP-10 and cathepsin F then delay wound healing [89–91]. Therefore, silencing of MMP-10 and cathepsin F in the diabetic cornea represents a potential therapeutic strategy to promote healing of the human CE ex vivo [89]. In addition, upon activation by the PI3K/AKT cascade, MMP-9 promotes the migration of human corneal limbal epithelial cells in vitro, and this process has the potential to accelerate healing of the CE [92]. Moreover, hyperglycaemia suppresses the expression of the antimicrobial peptide LL-37, which interacts with G protein-coupled receptors and transactivates EGFR, thus enhancing healing of the CE [93, 94].

Clinically, growth factors, NK-1, and MMPs have been widely used to treat DK. We discussed that PI3K/AKT signalling is a target pathway related to DK, indicating that other drugs that reactivate PI3K/AKT signalling can be developed to treat DK.

### DED and hyperosmolarity inhibit PI3K/AKT signalling to induce inflammation and apoptosis

DED-induced tear film hyperosmolarity inhibits the PI3K/AKT signalling, which is partially responsible for the occurrence and progression of inflammation, autophagy, and apoptosis in CECs [95].

Hyperosmolarity may increase the expression of high-mobility group box 1 (HMGB1), tumour necrosis factor α (TNF-α), and IL-1β in a dose-dependent manner by inhibiting PI3K/AKT signalling in CECs in vivo and in vitro [96]. Actually, HMGB1 causes a vicious proinflammatory cycle, maintains immune responses, and participates in tissue damage [97].

Therefore, reactivation of PI3K/AKT signalling may be an effective treatment for DED. As a type of carotene, astaxanthin restores the activity of the PI3K/AKT signalling pathway to promote HMGB1 expression and inflammation [96]. In addition, an immunodepressant, cyclosporine A (CsA), inhibits the expression of TNF-α, Bcl-2-associated X, and Bcl-2 and reduces apoptosis and inflammation by reactivating the PI3K/AKT signalling pathway in CECs in vitro [98, 99]. A previous study reported that CsA upregulates TGF-β1 expression in vivo [100]. Further studies are required to investigate the aforementioned paradox.

Furthermore, in an in vitro CEC model of DED, the transcription factor EB activates autophagy, and the PI3K/AKT signalling pathway inhibits transcription factor EB activity to exert an anti-autophagy effect [73]. However, a disaccharide, trehalose may inhibit PI3K/AKT signalling and increase the function of the transcription factor EB, thus increasing autophagy in CECs in vitro [73, 101]. Autophagy maintains a good balance of CEC inflammation by recycling macromolecules and thus restoring internal environmental balance [102, 103].

In summary, CsA, astaxanthin, and trehalose inhibit inflammation, apoptosis, and/or autophagy in DED models by restoring PI3K/AKT signalling pathway activity. Clinically, we propose that PI3K/AKT signalling may be a crucial target for DED treatment.

### Keratitis induces inflammation partially through the overactivation of PI3K/AKT signalling

The PI3K/AKT signalling pathway is a key pathway that mediates keratitis and has potential as a therapeutic target for keratitis.

#### Infectious keratitis

Infectious keratitis is mostly caused by microbial infections, such as bacterial, fungal, and viral infections. Studies have shown an important role the PI3K/AKT signalling pathway plays in the infection of CECs with these pathogens and is a target of many drugs.

Bacterial keratitis accounts for approximately 65–90% of all cases of microbial keratitis [104]. Bacteria induce inflammation in the CE through bacterial lipopolysaccharide (LPS), which increases HMGA2/PI3K/AKT signalling in vitro [18]. Activation of PI3K/AKT signalling then supports inflammation and the release of inflammatory cytokines such as TNF-α, IL-6, and IL-1β [105]. Importantly, some potential therapeutic drugs, such as cyanidin-3-O-glucoside (C3G) and asiatic acid, inhibit PI3K/AKT signalling to counteract the effects of LPS in vitro [18, 23]. In addition, bacterial infection may activate EGFR/PI3K/AKT signalling through the shedding of the HB-EGF extracellular domain, which prevents CEC apoptosis in the early stage of infection in vitro [50].

Fungal keratitis is caused by fungal infections, such as ***Fusarium***, ***Aspergillus***, and ***Candida***. Previous studies have shown that extracellular polysaccharide EPS-II partially inhibits the adherence of ***Candida albicans*** to CECs, partially suppresses PI3K/AKT signalling, and decreases levels of inflammatory proteins (IL-6 and MMP-14) in fungal keratitis [106, 107]. EPS-II, which competitively inhibits the adherence of ***C. albicans*** to hCECs and may activate cellular responses by binding with receptors, contains ligands similar to ***C. albicans*** in vitro [106].

Viral keratitis often causes infectious blindness, and herpes simplex virus type 1 (HSV-1) is one of the most common human pathogens [108]. Most of the current drugs used to treat HSV are nucleoside analogues, which have a high incidence of side effects and target DNA replication. Fortunately, recent studies have identified several promising and safer drugs that target the AKT signalling pathway to inhibit HSV infection in the cornea, including the natural secondary metabolite prodigiosin (PD), which is produced by ***Serratia marcescens***, and a selective 3-phosphoinositide-dependent kinase 1 inhibitor called BX795 [108, 109]. And the inhibitory effect of PD on HSV-1 in vivo, in vitro, and ex vivo, with positive therapeutic results observed in mouse models. PD treatment may inhibit the phosphorylation of AKT in CECs, preventing it from inactivating GSK3β, blocking apoptosis, promoting protein synthesis, and creating a host environment that facilitates viral replication [109]. Moreover, BX795 inhibits the PI3K/AKT/mTOR pathway and then blocks the hyperphosphorylation of eukaryotic initiation factor 4E-binding protein 1, which is a member of a family of translation repressor proteins [110, 111]. Hence, the virus cannot use the protein translation machinery of the host cells [111].

In summary, inflammation and virus replication are mediated by PI3K/AKT signalling in infectious keratitis. C3G, asiatic acid, EPS-II, PD, BX795, and other drugs reverse overactivation of PI3K/AKT signalling to treat infectious keratitis.

#### Noninfectious keratitis

Noninfectious keratitis is associated with injury and other inflammatory agonists. The PI3K/AKT signalling pathway is closely related to noninfectious keratitis, but the specific mechanism must be further elucidated. For instance, exposure to nitrogen mustard, a vesicating agent, increases cyclooxygenase 2 (an inflammation mediator), MMP-9, and vascular endothelial growth factor levels in CECs potentially through the activation of the AKT-activator protein 1 pathway in vitro and ex vivo [112]. Moreover, released from injured and inflammatory CECs, lysophosphatidic acid (LPA) induces transactivation of EGFR, and activation of PI3K and mediates inflammation by activating the production of inflammatory mediators, such as LPS, IL-1β, and TNF-α in CECs [113, 114]. We speculate that inhibition of the aforementioned inflammatory factors has therapeutic potential.

## Conclusions and perspectives

Overall, our review mainly summarizes the role of the PI3K/AKT signalling pathway in CECs. Growth factors (EGFR ligands, IGF1, etc.), NK-1, and insulin activate the PI3K/AKT signalling pathway by binding to their receptors, which inhibit apoptosis and inflammation, promote the proliferation and migration of CECs, and accelerate healing of the CE. Extreme environments-induced excess accumulation of ROS inhibits PI3K/AKT signaling, thus inducing CEC apoptosis and inflammation, but low or moderate levels of ROS activate PI3K/AKT inhibit apoptosis, and promote the healthy CEC migration and proliferation.

**Fig. 4:**
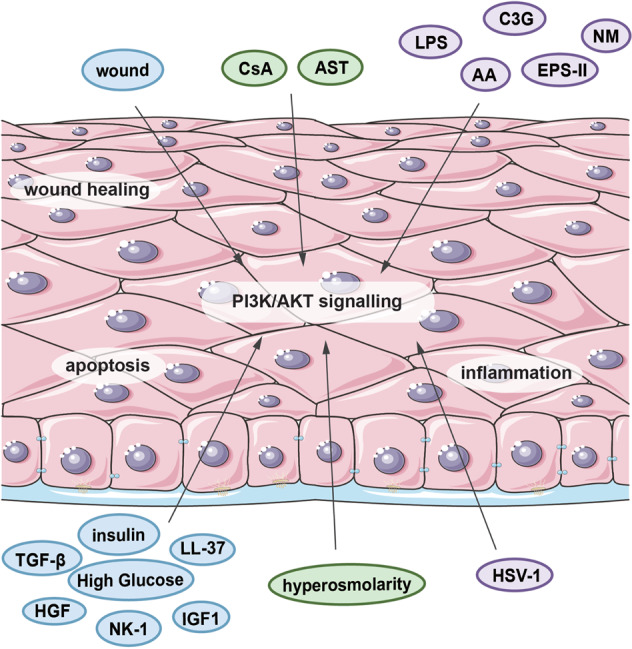
The localization and function of different molecules involved in the PI3K/AKT signalling pathway in the CE. At the tissue level, different molecules affect CECs by modulating the PI3K/AKT signalling pathway in vivo and in vitro. Some factors are extracellular, such as wounds, some drugs (AST, CsA, AA, and C3G), and some biological or chemical stimulants (LPS, EPS, and NM). Moreover, some factors exist in cells, such as glucose, insulin, various growth factors related to hyperglycaemia, and hyperosmolarity, which is related to DED.

Diabetes-associated hyperglycaemia directly inhibit PI3K/AKT signalling by increasing ROS and ER stress levels or suppressing receptors of growth factors in CECs and partially induce DK. Similarly, in DED, the PI3K/AKT signalling pathway is suppressed by hyperosmolar conditions and ROS. However, in keratitis, overactivation of the PI3K/AKT signalling pathway is responsible for inflammation and virus replication in CECs (Fig. 4).

We realized that a few studies have reported the status of PI3K/AKT signalling in different keratitis, but the specific mechanisms are not well known. Furthermore, the development of more effective drugs that target the PI3K/AKT signalling to treat related corneal diseases is needed.

## Supplementary information

Main text and tables (marked)-R3 with Data availability

Title, running title and abstract-R3

aj-checklist for CDDIS-22-0330R

Editing Certificate of AJE

## Data Availability

All relevant data are included in this manuscript.
