# Supplementary material for: The role of the PI3K/AKT signalling pathway in the corneal epithelium: recent updates
Source: Cell Death Dis. 2022 May 31;13(5):513. doi: 10.1038/s41419-022-04963-x (PMC9156734; doi:10.1038/s41419-022-04963-x)
Supplement: Supplementary file 1 — Main text and tables (marked)-R3 with Data availability [file 41419_2022_4963_MOESM1_ESM.docx]

**The role of the PI3K/AKT signalling pathway in the corneal epithelium: Recent updates.**

Kuangqi Chen^^[[1]](#footnote-1)^^, Yanqing Li^1^, Xuhong Zhang^1^, Rahim Ullah^2^, Jianping Tong^1^ and Ye Shen^1^

Correspondence: Ye Shen ([idrshen@zju.edu.cn](mailto:idrshen@zju.edu.cn))

Jianping Tong (idrtong@zju.edu.cn)

^1^ Department of Ophthalmology, the First Affiliated Hospital, School of Medicine, Zhejiang University, Hangzhou, zhejiang, 310003, PR China; Kuangqi Chen ([3160105142@zju.edu.cn](mailto:3160105142@zju.edu.cn)); Yanqing Li (3180103182@zju.edu.cn); Xuhong Zhang (11918408@zju.edu.cn);

^2^ Department of Endocrinology, Children's Hospital of Zhejiang University School of Medicine, National Clinical Research Center for Child Health, Hangzhou, Zhejiang, 310052, PR China; Rahim Ullah ([Rahim@zju.edu.cn](mailto:Rahim@zju.edu.cn));

*These authors contributed equally: Kuangqi Chen, Yanqing Li

**Abstract:** Phosphatidylinositol 3 kinase (PI3K)/AKT (also called protein kinase B, PKB) signalling regulates various cellular processes, such as apoptosis, cell proliferation, the cell cycle, protein synthesis, glucose metabolism, and telomere activity. Corneal epithelial cells (CECs) are the outermost cells of the cornea; they maintain good optical performance and act as a physical and immune barrier. Various growth factors, including epidermal growth factor receptor (EGFR) ligands, insulin-like growth factor 1 (IGF1), neurokinin 1 (NK-1), and insulin activate the PI3K/AKT signalling pathway by binding their receptors and promote antiapoptotic, anti-inflammatory, proliferative, and migratory functions and wound healing in the corneal epithelium (CE). Reactive oxygen species (ROS) regulate apoptosis and inflammation in CECs in a concentration-dependent manner. Extreme environments induce excess ROS accumulation, inhibit PI3K/AKT, and cause apoptosis and inflammation in CECs. However, at low or moderate levels, ROS activate PI3K/AKT signalling, inhibiting apoptosis and stimulating proliferation of healthy CECs. Diabetes-associated hyperglycaemia directly inhibit PI3K/AKT signalling by increasing ROS and endoplasmic reticulum (ER) stress levels or suppressing the expression of growth factors receptors and cause diabetic keratopathy (DK) in CECs. Similarly, hyperosmolarity and ROS accumulation suppress PI3K/AKT signalling in dry eye disease (DED). However, significant overactivation of the PI3K/AKT signalling pathway, which mediates inflammation in CECs, is observed in both infectious and noninfectious keratitis. Overall, upon activation by growth factors and NK-1, PI3K/AKT signalling promotes the proliferation, migration, and anti-apoptosis of CECs, and these processes can be regulated by ROS in a concentration-dependent manner. Moreover, PI3K/AKT signalling pathway is inhibited in CECs from individuals with DK and DED, but is overactivated by keratitis.

**Keywords:** PI3K, AKT, corneal epithelial cells, corneal epithelium, wound healing

**Facts**

CECs are present in the outermost layer of the cornea and play an important role in maintaining CE homeostasis and optical function.

The PI3K/AKT signalling pathway is widely involved in the proliferation, apoptosis, migration, and other functions of CECs.

Many corneal diseases and wound healing are associated with the PI3K/AKT signalling and its interaction with ROS.

**Open questions**

How do CECs respond to different environmental conditions, such as health and disease states, through the PI3K/AKT signalling pathway?

How does the PI3K/AKT signalling pathway interact with ROS in CECs to control the cellular response and influence disease progression?

Can the PI3K/AKT signalling pathway be used as a major target for the treatment of corneal diseases in the future?

**1. Introduction**

In 1987, Staal first discovered a proto-oncogene, i.e., the *AKT1* gene on human chromosome 14, band q32 [1, 2]. In subsequent years, a lot of studies gradually revealed the intracellular signal transduction cascade centred on phosphatidylinositol 3 kinase (PI3K) and AKT, named the PI3K/AKT signalling pathway. The process of the PI3K/AKT signalling pathway mainly includes binding of exogenous factors to receptors, receptor activation of PI3K phosphorylation, PI3K-induced phosphorylation of AKT, and initiation of downstream effectors [3]. Activated PI3K further catalyses the production of the second messenger phosphatidylinositol-3,4,5-triphosphate (PIP3). The binding of PIP3 to the PH domain and plasma membrane translocation of AKT trigger its phosphorylation [4]. After that, PI3K/AKT activates the phosphorylation or complex formation of downstream molecules, including glycogen synthase kinase 3β (GSK3β), mammalian target of rapamycin (mTOR), and actin-related proteins [5, 6]. These molecules then regulate metabolic functions, such as gluconeogenesis, protein synthesis, cell cycle, migration and apoptosis [7].

The eye is a complex structure that consists of the cornea, lens, vitreous, choroid, retina, optic nerve, and other accessory structures (Figure 1). The cornea is composed of the corneal epithelium (CE), Bowman's layer, corneal stroma, Descemet's membrane and the endothelium. The CE is a self-renewing stratified nonkeratinized squamous epithelium that protects the inner eye tissue, forms the immune barrier, supports the tear film, and maintains transparency [8, 9]. Corneal epithelial cells (CECs) include superficial squamous cells, central suprabasal cells, and a single layer of inner columnar basal cells [10]. During CE homeostasis, the adult CE is maintained by a comprehensive process involving cell proliferation, migration, differentiation and apoptosis. CECs undergo inflammation, apoptosis and/or autophagy when the CE is damaged, and the adjacent basal cells of the CE secrete a large number of cytokines, growth factors to promote proliferation, differentiation and migration, ultimately achieving wound healing by regulating signalling networks in CECs [11].

Diabetic keratopathy (DK) is caused by diabetes-associated hyperglycaemia (a persistent high-glucose state in blood in general) and manifests as persistent corneal epithelial erosion, superficial punctate keratopathy, delayed epithelial regeneration, and reduced corneal sensitivity, which may cause impaired vision or permanent vision loss [12]. Dry eye disease (DED) is a multifactorial and chronic disease characterized by tear deficiency and/or evaporative dry eye, and causes the tear film covering the CE to adopt a local hyperosmotic state, which causes damage to the CE and delayed wound healing [13]. Keratitis is a general term describing inflammatory damage to the cornea resulting from various causes, such as microbial infection, neurotrophin deficiency, trauma, and autoimmune disorders. Infectious keratitis refers to keratitis caused by microbial infection, and keratitis from other causes is called noninfectious keratitis. Corneal epithelial defects and delayed wound healing caused by inflammation are important clinical manifestations of keratitis [14, 15].

The PI3K/AKT signalling pathway plays an important role in the biological responses of CECs [16-19]. Importantly, dysregulation of CE homeostasis caused by impaired CEC responses is associated with many corneal diseases, such as DK, DED, and keratitis [18, 20, 21]. Therefore, an in-depth understanding of the pathophysiology of CE with a focus on the PI3K/AKT signalling pathway is important for maintaining the integrity of patients' corneas and seeking reliable treatments and new drugs [22, 23]. In this review, we provide evidence for the regulatory role of the PI3K/AKT signalling pathway in CECs to support its clinical application.

**2. Methods**

We searched the PubMed database (https://pubmed.ncbi.nlm.nih.gov/) using the keywords “AKT AND corneal epithelial cells” and “AKT AND corneal epithelium”. Afterwards, we carefully read and categorized the results, removed some irrelevant articles, and then focused on recent studies and reviews (published between 2015 and 2022). Additional studies were discovered by consulting the reference lists of the selected articles. Most of the references cited in this review are experimental articles, although a few review articles, case reports, and book chapters are included; no other types of literature are cited. Our figures were edited with Adobe Illustrator CC 2018 software (Adobe, San Jose, CA).

**3. Roles of growth factors and other hormones in the regulation of CEC functions**

Growth factors and hormones activate PI3K/AKT signalling in CECs, which regulates apoptosis, proliferation, migration, and wound healing (Table 1, Figure 2).

**3.1** **Epidermal growth factor receptor (EGFR) ligands**

Through matrix metalloproteinase (MMP)-dependent ectodomain shedding and transactivation of EGFR signalling (proto-oncogene tyrosine-protein kinase Src/PI3K/AKT), EGFR ligands, including epidermal growth factor (EGF), transforming growth factor-α, and heparin-binding EGF-like growth factor (HB-EGF), inhibit apoptosis and promote proliferation, migration in CECs, and healing of the CE [24].

Eukaryotic translation initiation factor 5A (eIF5A), a nucleocytoplasmic shuttle protein, is the substrate of the EGF-induced EGFR/Src/PI3K/AKT/mTOR/p70S6K/rpS6 signalling pathway and induces the expression of MMP-9 in the extracellular matrix and proliferating cell nuclear antigen *in vitro* [25, 26]. Furthermore, in mouse CECs *in vivo*, excess transforming growth factor-α expression promotes the expression of proliferating cell nuclear antigen likely by transactivating the EGFR/PI3K/AKT cascade. MMP-9 mediates the ectodomain shedding of EGF and/or HB-EGF, further activating EGFR/Src/PI3K/AKT signalling by increasing local proteolysis. Proliferating cell nuclear antigen facilitates proliferation by inducing DNA duplication through an increase the activity of DNA polymerase and eIF5A [25, 26].

The HB-EGF/EGFR/Src cascade is a target of various endogenous and exogenous molecules. Furthermore, the influx of extracellular Ca^2+^ increases the activity of Src, and a disintegrin and metalloproteinase and transactivating the HB-EGF/EGFR/Src/PI3K/AKT cascade [27, 28]. In addition, endogenous anti-inflammatory mediators, resolvins, induce MMP-mediated activation of the HB-EGF/EGFR/PI3K/AKT/GSK3β cascade, which inactivates paxillin to inhibit the activation of its substrate focal adhesion kinase and induce the formation of the mitogen-activated protein kinase 3/1-focal adhesion kinase-paxillin complex [29, 30]. These effects increase the migration of human CECs (hCECs) and healing of the CE *in vitro* [29, 30].

The endocannabinoid system regulates the proliferation and migration of CECs partially by transactivating or inhibiting the EGFR/PI3K/AKT signalling pathway [31, 32]. Two endogenous metabolites, capsaicin and MAP kinase kinase kinase win, and their receptors, cannabinoid receptor 1 and transient receptor potential vanilloid 1, respectively, were identified in previous studies. Activated cannabinoid receptor 1 and transient receptor potential vanilloid 1 increase the migration and proliferation partially through Ca^2+^ influx-induced transactivation of EGFR/Src/PI3K/AKT signalling in hCECs and mouse CECs *in vitro* [32, 33]. However, in bovine CECs *in vitro*, endogenous activation of cannabinoid receptor 1 antagonizes the transactivation of the EGFR/PI3K/AKT signalling pathway and promotes migration by inducing chemotaxis rather than inducing proliferation [34]. This opposite outcome may be attributed to the differences among humans, mice, and bovines, and we propose that related experiments on the corneas of other species are needed to further explore the effect of the cannabinoid system on the EGFR/PI3K/AKT cascade in CECs. More importantly, further *in vivo* experiments on the endocannabinoid system-EGFR/PI3K/AKT cascade are also necessary.

**3.2 Insulin-like growth factor 1 (IGF1) and insulin**

In hCECs, IGF1 binds to the IGF1 receptor to activate the insulin receptor substrate 2/PI3K/AKT signalling pathway and activate proliferation, but these effects are inhibited by IGF-binding protein 3 in CECs [35-38]. Furthermore, GSK3β may be phosphorylated by the insulin/insulin receptor /insulin receptor substrate 2/PI3K/AKT signalling cascade to induce degradation of the insulin receptor voltage-dependent anion channel-1 complex at the mitochondrial membrane, leading to senescence, mitophagy, and CEC survival *in vitro* [39].

Other growth factors, including transforming growth factor-β (TGF-β), hepatocyte growth factor (HGF), keratinocyte growth factor, nerve growth factor, vascular endothelial growth factor B, and pigment epithelium-derived factor, also activate the PI3K/AKT signalling, which promotes the proliferation, migration of CECs, inhibits CEC apoptosis and promotes healing of the CE [40-44].

The relationship between growth factors and DK is discussed in section 5.2.

**3.3 Neurokinin 1 (NK-1)**

As a neuropeptide that is widely distributed in nerve fibres, NK-1 (also called substance P) facilitate healing in the CE and the recovery of mitochondrial function in CECs [45, 46]. By binding to the NK-1 receptor, NK-1 restores EGFR/AKT/GSK3β signalling, which eliminates Caspase-3 (Cas-3) and reactive oxygen species (ROS) to inhibit CEC apoptosis under hyperglycaemic and hyperosmolar conditions [45, 47-50]. Cas-3 with reduced expression and activity cannot execute apoptosis in cytosol [50]. In addition, activation of AKT signalling partially inhibits the production of interleukin (IL)-1α, macrophage inflammatory protein 1α, and macrophage inflammatory protein 1β, all of which are associated with local inflammation and delayed healing of the CE [48]. Moreover, the NK-1 and C domains of IGFs synergistically activate the PI3K/AKT/GSK3β signalling pathway to promote cell migration [38, 51, 52].

**4. Interactions between ROS and AKT in CECs**

ROS are the byproducts of oxygen and are produced due to cellular metabolism, and regulate the redox balance, proliferation, cytotoxicity, and metabolic adaptation in a concentration-dependent manner [47, 53-56]. In the CE, ROS production is triggered by damage outside the eye (including chemical- or UV light-induced damage, and mechanical damage), as well as by inflammatory factors in the aqueous humour or tears [57-59].

**4.1 Extreme environments-induced excessive accumulation of ROS**

Extreme environments including hyperglycaemic and hyperosmolar environments may cause excess ROS accumulation, which is considered an upstream suppressor of the EGFR-mediated PI3K/AKT signalling pathway, and delays healing of the CE [60]. *In vitro* and *in vivo* studies have shown that inhibition of ROS production reactivates PI3K/AKT signalling, which decreases apoptosis and inflammation in CECs [22, 23]. Furthermore, the expression of the cellular tumour antigen P53, a PI3K/AKT substrate, is upregulated by ROS in hCECs [61, 62]. In this manner, ROS increase Bcl-2, Bcl-2-associated X, and cytochrome c levels in mitochondria, and Cas-3, Cas-8, and Cas-9 levels in the cytosol and cleave poly (ADP-ribose) polymerase in the nucleus. These proteins are all substrates of PI3K/AKT signalling and apoptosis-related proteins [61, 63].

The role of ROS in diabetes and hyperglycaemia is discussed in section 5.1.1.

**4.2 Low or moderate levels of ROS**

Interestingly, ROS production may also be induced by growth factors, and may function as a second messenger to activate PI3K/AKT signalling at low or moderate levels. These effects stimulate the proliferation and migration of healthy CECs, inhibit apoptosis of healthy CECs, and induce healing of the CE *in vitro* (in human and rabbit CECs) and *ex vivo* (in pig cornea) [64, 65]. Although no studies have investigated the targets of the AKT signalling pathway in CECs, a study in another cell type indicated that the possible targets of ROS in the PI3K/AKT signalling pathway include phosphatase and tensin homologue, the P85 domain of PI3K [66]. Phosphatase and tensin homologue impairs PI3K/AKT signalling via the hydrolysis of phosphorylation sites of PI3K, PIP2, and PIP3, resulting in delayed proliferation and migration of CECs [67]. Studies in CECs and lens epithelial cells suggest that the production of very low levels of ROS may be triggered by growth factors and produced by NADPH oxidase in the plasma membrane rather than mitochondria to activate PI3K/AKT signalling, however, this result requires further confirmation in CECs [64, 68-70].

**5. Diseases, their therapies, and AKT in CECs**

In the healthy CE, the levels of phosphorylated PI3K and AKT are relatively low to maintain the homeostasis of CECs. When the CE is damaged, growth factors and NK-1 are secreted to activate the PI3K/AKT cascade, which promotes proliferation and migration and inhibits apoptosis and inflammation of CECs. These effects together enable the healing of the CE. However, under the influence of various factors (such as hyperglycaemia, hyperosmolarity, excess inflammatory factors and cytokines, toxicants, and microbial infection), both the CE homeostasis and the normal expression of the PI3K/AKT signalling pathway are disrupted. These processes are associated with many diseases, including DK, DED, and keratitis (Table 2, Figure 3). Hence, molecules targeting the PI3K/AKT signalling pathway have become promising therapies [22, 71-73].

**5.1 Diabetes-associated hyperglycaemia inhibits PI3K/AKT signalling to induce apoptosis and inflammation, and delay wound healing**

Diabetes-associated hyperglycaemia stimulates apoptosis and inflammation but inhibits migration and tight junctions in CECs, thus causing DK [20].

**5.1.1 Hyperglycaemia-induced excess ROS accumulation inhibits PI3K/AKT signalling to increase apoptosis and delay wound healing**

Hyperglycaemic conditions induce mitochondrial fragmentation, which leads to ROS overproduction that inactivates PI3K/AKT [74]. Silent information regulator 1 (SIRT1) is a substrate of PI3K/AKT signalling that decreases ROS production, apoptosis, and endoplasmic reticulum (ER) stress in CECs *in vitro* [75]. Hyperglycaemia reduces SIRT1 expression, increases P53 acetylation, and inhibits the IGF1/PI3K/AKT cascade in CECs, which may cause delayed healing of the CE *in vivo* and *in vitro* [76, 77].

Antioxidant therapy, which induces the clearance of excess ROS and activates the AKT pathway, is an effective drug treatment strategy for DK. For example, N-acetylcysteine reactivates the PI3K/AKT signalling pathway by inhibiting excess ROS production and restoring CEC migration and healing of the diabetic human and pig CE *in vitro* and *ex vivo* [60]. Similarly, exogenous NAD^+^ and its precursors nicotinamide mononucleotide and nicotinamide riboside attenuate ROS accumulation, restore the mitochondrial membrane potential and facilitate the healing of the CE and regeneration of corneal nerves [77]. Moreover, NK-1 repairs mitochondrial damage and clears accumulated ROS, thus improving diabetic CE wound healing and corneal sensation [45, 78, 79].

**5.1.2 Hyperglycaemia-induced ER stress suppresses AKT to inhibit wound healing.**

ER stress inhibits the PI3K/AKT signalling pathway and causes apoptosis and inflammation [80]. In individuals with DK, an imbalance in ROS levels causes ER stress [81, 82]. A study examining diabetic rabbit CECs found that hyperglycaemia induces the expression of protein kinase RNA-like ER kinase and C/EBP-homologous protein, inducing ER stress and subsequently inhibiting the AKT signalling pathway in activate apoptosis *in vitro* [74].

Interestingly, inhibition of ER stress activates the AKT pathway and promotes healing of the CE. As neuroprotective factors, most mesencephalic astrocyte-derived neurotrophic factors are retained in the ER and play an important role in maintaining ER stability [83, 84]. Moreover, mesencephalic astrocyte-derived neurotrophic factors inhibit hyperglycaemia-induced ER stress, which promotes wound healing and nerve regeneration in the CE under normal and diabetic conditions through AKT activation [85].

**5.1.3 Hyperglycaemia-induced inactivation of growth factor receptors inhibits PI3K/AKT signalling, increases apoptosis, and delays wound healing**

Hyperglycaemia inhibits the expression of growth factor receptors in CECs. In the context of diabetes, the expression of the c-met (also called HGF receptor), which may directly inhibit HGF/c-met/PI3K/AKT signalling, is decreased in CECs *in vitro* [86]. The expression and phosphorylation/activity of c-met are restored by recombinant adenovirus-driven c-met overexpression, which normalizes wound healing and the expression of diabetes markers in diabetic hCECs [86]. This effect may be explained by the interactions between the HGF/c-met system and other growth factors including IGF and EGF [87, 88].

Inactivation of the EGFR/PI3K/AKT signalling pathway leads to the overexpression of MMP-10 and cathepsin F. MMP-10 and cathepsin F then delay wound healing [89-91]. Therefore, silencing of MMP-10 and cathepsin F in the diabetic cornea represents a potential therapeutic strategy to promote healing of the human CE *ex vivo* [89]. In addition, upon activation by the PI3K/AKT cascade, MMP-9 promotes the migration of human corneal limbal epithelial cells *in vitro*, and this process has the potential to accelerate healing of the CE [92]. Moreover, hyperglycaemia suppresses the expression of the antimicrobial peptide LL-37, which interacts with G protein-coupled receptors and transactivates EGFR, thus enhancing healing of the CE [93, 94].

Clinically, growth factors, NK-1, and MMPs have been widely used to treat DK. We discussed that PI3K/AKT signalling is a target pathway related to DK, indicating that other drugs that reactivate PI3K/AKT signalling can be developed to treat DK.

**5.2 DED and hyperosmolarity inhibit PI3K/AKT signalling to induce inflammation and apoptosis**

DED-induced tear film hyperosmolarity inhibits the PI3K/AKT signalling, which is partially responsible for the occurrence and progression of inflammation, autophagy, and apoptosis in CECs [95].

Hyperosmolarity may increase the expression of high-mobility group box 1 (HMGB1), tumour necrosis factor α (TNF-α), and IL-1β in a dose-dependent manner by inhibiting PI3K/AKT signalling in CECs *in vivo* and *in vitro* [96]. Actually, HMGB1 causes a vicious proinflammatory cycle, maintains immune responses, and participates in tissue damage [97].

Therefore, reactivation of PI3K/AKT signalling may be an effective treatment for DED. As a type of carotene, astaxanthin restores the activity of the PI3K/AKT signalling pathway to promote HMGB1 expression and inflammation [96]. In addition, an immunodepressant, cyclosporine A (CsA), inhibits the expression of TNF-α, Bcl-2-associated X, and Bcl-2 and reduces apoptosis and inflammation by reactivating the PI3K/AKT signalling pathway in CECs *in vitro* [98, 99]. A previous study reported that CsA upregulates TGF-β1 expression *in vivo* [100]. Further studies are required to investigate the aforementioned paradox.

Furthermore, in an *in vitro* CEC model of DED, the transcription factor EB activates autophagy, and the PI3K/AKT signalling pathway inhibits transcription factor EB activity to exert an anti-autophagy effect [73]. However, a disaccharide, trehalose may inhibit PI3K/AKT signalling and increase the function of the transcription factor EB, thus increasing autophagy in CECs *in vitro* [73, 101]. Autophagy maintains a good balance of CEC inflammation by recycling macromolecules and thus restoring internal environmental balance [102, 103].

In summary, CsA, astaxanthin, and trehalose inhibit inflammation, apoptosis, and/or autophagy in DED models by restoring PI3K/AKT signalling pathway activity. Clinically, we propose that PI3K/AKT signalling may be a crucial target for DED treatment.

**5.3 Keratitis induces inflammation partially through the overactivation of PI3K/AKT signalling**

The PI3K/AKT signalling pathway is a key pathway that mediates keratitis and has potential as a therapeutic target for keratitis.

**5.3.1 Infectious keratitis**

Infectious keratitis is mostly caused by microbial infections, such as bacterial, fungal, and viral infections. Studies have shown an important role the PI3K/AKT signalling pathway plays in the infection of CECs with these pathogens and is a target of many drugs.

Bacterial keratitis accounts for approximately 65–90% of all cases of microbial keratitis [104]. Bacteria induce inflammation in the CE through bacterial lipopolysaccharide (LPS), which increases HMGA2/PI3K/AKT signalling *in vitro* [18]. Activation of PI3K/AKT signalling then supports inflammation and the release of inflammatory cytokines such as TNF-α, IL-6, and IL-1β [105]. Importantly, some potential therapeutic drugs, such as cyanidin-3-O-glucoside (C3G) and asiatic acid, inhibit PI3K/AKT signalling to counteract the effects of LPS *in vitro* [18, 23]. In addition, bacterial infection may activate EGFR/PI3K/AKT signalling through the shedding of the HB-EGF extracellular domain, which prevents CEC apoptosis in the early stage of infection *in vitro* [50].

Fungal keratitis is caused by fungal infections, such as ***Fusarium, Aspergillus,*** and ***Candida***. Previous studies have shown that extracellular polysaccharide EPS-II partially inhibits the adherence of ***Candida albicans*** to CECs, partially suppresses PI3K/AKT signalling, and decreases levels of inflammatory proteins (IL-6 and MMP-14) in fungal keratitis [106, 107]. EPS-II, which competitively inhibits the adherence of ***C. albicans*** to hCECs and may activate cellular responses by binding with receptors, contains ligands similar to ***C. albicans*** *in vitro* [106].

Viral keratitis often causes infectious blindness, and herpes simplex virus type 1 (HSV-1) is one of the most common human pathogens [108]. Most of the current drugs used to treat HSV are nucleoside analogues, which have a high incidence of side effects and target DNA replication. Fortunately, recent studies have identified several promising and safer drugs that target the AKT signalling pathway to inhibit HSV infection in the cornea, including the natural secondary metabolite prodigiosin (PD), which is produced by ***Serratia marcescens***, and a selective 3-phosphoinositide-dependent kinase 1 inhibitor called BX795 [108, 109]. And the inhibitory effect of PD on HSV-1 *in vivo*, *in vitro*, and *ex vivo*, with positive therapeutic results observed in mouse models. PD treatment may inhibit the phosphorylation of AKT in CECs, preventing it from inactivating GSK3β, blocking apoptosis, promoting protein synthesis, and creating a host environment that facilitates viral replication [109]. Moreover, BX795 inhibits the PI3K/AKT/mTOR pathway and then blocks the hyperphosphorylation of eukaryotic initiation factor 4E-binding protein 1, which is a member of a family of translation repressor proteins [110, 111]. Hence, the virus cannot use the protein translation machinery of the host cells [111].

In summary, inflammation and virus replication are mediated by PI3K/AKT signalling in infectious keratitis. C3G, asiatic acid, EPS-II, PD, BX795, and other drugs reverse overactivation of PI3K/AKT signalling to treat infectious keratitis.

**5.3.2 Noninfectious keratitis**

Noninfectious keratitis is associated with injury and other inflammatory agonists. The PI3K/AKT signalling pathway is closely related to noninfectious keratitis, but the specific mechanism must be further elucidated. For instance, exposure to nitrogen mustard, a vesicating agent, increases cyclooxygenase 2 (an inflammation mediator), MMP-9, and vascular endothelial growth factor levels in CECs potentially through the activation of the AKT-activator protein 1 pathway *in vitro* and *ex vivo* [112]. Moreover, released from injured and inflammatory CECs, lysophosphatidic acid (LPA) induces transactivation of EGFR, and activation of PI3K and mediates inflammation by activating the production of inflammatory mediators, such as LPS, IL-1β, and TNF-α in CECs [113, 114]. We speculate that inhibition of the aforementioned inflammatory factors has therapeutic potential.

**6. Conclusions and Perspectives**

Overall, our review mainly summarizes the role of the PI3K/AKT signalling pathway in CECs. Growth factors (EGFR ligands, IGF1, etc.), NK-1, and insulin activate the PI3K/AKT signalling pathway by binding to their receptors, which inhibit apoptosis and inflammation, promote the proliferation and migration of CECs, and accelerate healing of the CE. Extreme environments-induced excess accumulation of ROS inhibits PI3K/AKT signaling, thus inducing CEC apoptosis and inflammation, but low or moderate levels of ROS activate PI3K/AKT inhibit apoptosis, and promote the healthy CEC migration and proliferation.

Diabetes-associated hyperglycaemia directly inhibit PI3K/AKT signalling by increasing ROS and ER stress levels or suppressing receptors of growth factors in CECs and partially induce DK. Similarly, in DED, the PI3K/AKT signalling pathway is suppressed by hyperosmolar conditions and ROS. However, in keratitis, overactivation of the PI3K/AKT signalling pathway is responsible for inflammation and virus replication in CECs (Figure 4).

We realized that a few studies have reported the status of PI3K/AKT signalling in different keratitis, but the specific mechanisms are not well known. Furthermore, the development of more effective drugs that target the PI3K/AKT signalling to treat related corneal diseases is needed.

**Data Availability**

All relevant data are included in this manuscript.

**Acknowledgements**

We would like to thank the authors of the primary studies. We used Adobe Illustrator to create the figures in this article.

**Funding**

This work was supported by the Strategic Priority Research Program of Chinese Academy of Sciences (Grant Number XDA16040200), the Natural Science Foundation of Zhejiang Province (Grant Number LZ19H120001), the Major Science and Technology Project of Zhejiang Province (Grant Number 2018C03017).

**Conflict of Interest**

The authors declare that no conflict of interest could be perceived as prejudicing the impartiality of the review.

**Author Contributions**

K.-Q.C. and Y.-Q.L. wrote the manuscript, Y.S., T.-J.P. X.-H.Z. and U.R. prepared the references and revision of the manuscript.

**Ethics Approval and Consent to Participate**

This research was based on the review of published/publicly reported literature and did not require ethical approval.

**References**

1. Staal SP. Molecular cloning of the akt oncogene and its human homologues AKT1 and AKT2: amplification of AKT1 in a primary human gastric adenocarcinoma. Proc Natl Acad Sci U S A. 1987;84(14):5034-7.

2. Staal SP, Huebner K, Croce CM, Parsa NZ, Testa JR. The AKT1 proto-oncogene maps to human chromosome 14, band q32. Genomics. 1988;2(1):96-8.

3. Manning BD, Toker A. AKT/PKB Signaling: Navigating the Network. Cell. 2017;169(3):381-405.

4. Masayuki N, Toshiyuki O, Futoshi S. Regulation of the PI3K-Akt Network: Current Status and a Promise for the Treatment of Human Diseases. Current Signal Transduction Therapy. 2008;3(2):138-51.

5. Matsuo FS, Andrade MF, Loyola AM, da Silva SJ, Silva MJB, Cardoso SV, et al. Pathologic significance of AKT, mTOR, and GSK3β proteins in oral squamous cell carcinoma-affected patients. Virchows Archiv : an international journal of pathology. 2018;472(6):983-97.

6. Mirdamadi Y, Bommhardt U, Goihl A, Guttek K, Zouboulis CC, Quist S, et al. Insulin and Insulin-like growth factor-1 can activate the phosphoinositide-3-kinase /Akt/FoxO1 pathway in T cells in vitro. Dermatoendocrinol. 2017;9(1):e1356518.

7. Franke TF. PI3K/Akt: getting it right matters. Oncogene. 2008;27(50):6473-88.

8. Yazdanpanah G, Jabbehdari S, Djalilian AR. Limbal and corneal epithelial homeostasis. Current opinion in ophthalmology. 2017;28(4):348-54.

9. Masterton S, Ahearne M. Mechanobiology of the corneal epithelium. Experimental Eye Research. 2018;177:122-9.

10. Secker GA, Daniels JT. Limbal epithelial stem cells of the cornea: Harvard Stem Cell Institute; 2009 2009/06/30.

11. Wilson SE. Corneal wound healing. Experimental eye research. 2020;197:108089.

12. Shah R, Amador C, Tormanen K, Ghiam S, Saghizadeh M, Arumugaswami V, et al. Systemic diseases and the cornea. Experimental eye research. 2021;204:108455.

13. Craig JP, Nichols KK, Akpek EK, Caffery B, Dua HS, Joo CK, et al. TFOS DEWS II Definition and Classification Report. The ocular surface. 2017;15(3):276-83.

14. Lakhundi S, Siddiqui R, Khan NA. Pathogenesis of microbial keratitis. Microbial pathogenesis. 2017;104:97-109.

15. Sharma S. Keratitis. Biosci Rep. 2001;21(4):419-44.

16. Guo Y, Wu W, Ma X, Shi M, Yang X. Comparative gene expression profiling reveals key pathways and genes different in skin epidermal stem cells and corneal epithelial cells. Genes & Genomics. 2019;41(6):679-88.

17. Han L, Su W, Huang J, Zhou J, Qiu S, Liang D. Doxycycline Inhibits Inflammation-Induced Lymphangiogenesis in Mouse Cornea by Multiple Mechanisms. PLOS ONE. 2014;9(9):e108931.

18. Li X, Sun M, Long Y. Cyanidin-3-O-Glucoside Attenuates Lipopolysaccharide-Induced Inflammation in Human Corneal Epithelial Cells by Inducing Let-7b-5p-Mediated HMGA2/PI3K/Akt Pathway. Inflammation. 2020;43(3):1088-96.

19. Bettahi I, Sun H, Gao N, Wang F, Mi X, Chen W, et al. Genome-Wide Transcriptional Analysis of Differentially Expressed Genes in Diabetic, Healing Corneal Epithelial Cells: Hyperglycemia-Suppressed TGFβ3 Expression Contributes to the Delay of Epithelial Wound Healing in Diabetic Corneas. Diabetes. 2014;63(2):715-27.

20. Liu F, Kong H, Kong X. Transforming growth factor-β blocks glucose-induced inflammation and apoptosis in corneal epithelial cells. FEBS open bio. 2018;8(12):1936-42.

21. Redfern RL, Barabino S, Baxter J, Lema C, McDermott AM. Dry eye modulates the expression of toll-like receptors on the ocular surface. Experimental eye research. 2015;134:80-9.

22. Park JH, Moon SH, Kang DH, Um HJ, Kang SS, Kim JY, et al. Diquafosol Sodium Inhibits Apoptosis and Inflammation of Corneal Epithelial Cells Via Activation of Erk1/2 and RSK: In Vitro and In Vivo Dry Eye Model. Invest Ophthalmol Vis Sci. 2018;59(12):5108-15.

23. Chen H, Hua XM, Ze BC, Wang B, Wei L. The anti-inflammatory effects of asiatic acid in lipopolysaccharide-stimulated human corneal epithelial cells. International journal of ophthalmology. 2017;10(2):179-85.

24. Peterson JL, Ceresa BP. Epidermal Growth Factor Receptor Expression in the Corneal Epithelium. Cells. 2021;10(9).

25. Ding L, Gao LJ, Gu PQ, Guo SY, Cai YQ, Zhou XT. The role of eIF5A in epidermal growth factor-induced proliferation of corneal epithelial cell association with PI3-k/Akt activation. Mol Vis. 2011;17:16-22.

26. Zhang L, Yuan Y, Yeh LK, Dong F, Zhang J, Okada Y, et al. Excess Transforming Growth Factor-α Changed the Cell Properties of Corneal Epithelium and Stroma. Invest Ophthalmol Vis Sci. 2020;61(8):20.

27. Yin J, Xu K, Zhang J, Kumar A, Yu FS. Wound-induced ATP release and EGF receptor activation in epithelial cells. J Cell Sci. 2007;120(Pt 5):815-25.

28. Klepeis VE, Weinger I, Kaczmarek E, Trinkaus-Randall V. P2Y receptors play a critical role in epithelial cell communication and migration. J Cell Biochem. 2004;93(6):1115-33.

29. Zhang F, Yang H, Pan Z, Wang Z, Wolosin JM, Gjorstrup P, et al. Dependence of resolvin-induced increases in corneal epithelial cell migration on EGF receptor transactivation. Invest Ophthalmol Vis Sci. 2010;51(11):5601-9.

30. Teranishi S, Kimura K, Nishida T. Role of formation of an ERK-FAK-paxillin complex in migration of human corneal epithelial cells during wound closure in vitro. Invest Ophthalmol Vis Sci. 2009;50(12):5646-52.

31. Nguyen AX, Wu AY. Cannabis and the Cornea. Ocular immunology and inflammation. 2021;29(5):1023-8.

32. Yang Y, Yang H, Wang Z, Varadaraj K, Kumari SS, Mergler S, et al. Cannabinoid receptor 1 suppresses transient receptor potential vanilloid 1-induced inflammatory responses to corneal injury. Cellular signalling. 2013;25(2):501-11.

33. Yang H, Wang Z, Capó-Aponte JE, Zhang F, Pan Z, Reinach PS. Epidermal growth factor receptor transactivation by the cannabinoid receptor (CB1) and transient receptor potential vanilloid 1 (TRPV1) induces differential responses in corneal epithelial cells. Experimental eye research. 2010;91(3):462-71.

34. Murataeva N, Li S, Oehler O, Miller S, Dhopeshwarkar A, Hu SS, et al. Cannabinoid-induced chemotaxis in bovine corneal epithelial cells. Invest Ophthalmol Vis Sci. 2015;56(5):3304-13.

35. Wu YC, Zhu M, Robertson DM. Novel nuclear localization and potential function of insulin-like growth factor-1 receptor/insulin receptor hybrid in corneal epithelial cells. PLoS One. 2012;7(8):e42483.

36. Robertson DM, Zhu M, Wu YC. Cellular distribution of the IGF-1R in corneal epithelial cells. Experimental eye research. 2012;94(1):179-86.

37. Rocha EM, Cunha DA, Carneiro EM, Boschero AC, Saad MJ, Velloso LA. Identification of insulin in the tear film and insulin receptor and IGF-1 receptor on the human ocular surface. Invest Ophthalmol Vis Sci. 2002;43(4):963-7.

38. Stuard WL, Titone R, Robertson DM. The IGF/Insulin-IGFBP Axis in Corneal Development, Wound Healing, and Disease. Front Endocrinol (Lausanne). 2020;11:24.

39. Titone R, Robertson DM. Insulin receptor preserves mitochondrial function by binding VDAC1 in insulin insensitive mucosal epithelial cells. Faseb j. 2020;34(1):754-75.

40. Chandrasekher G, Pothula S, Maharaj G, Bazan HE. Differential effects of hepatocyte growth factor and keratinocyte growth factor on corneal epithelial cell cycle protein expression, cell survival, and growth. Mol Vis. 2014;20:24-37.

41. Hong J, Qian T, Le Q, Sun X, Wu J, Chen J, et al. NGF promotes cell cycle progression by regulating D-type cyclins via PI3K/Akt and MAPK/Erk activation in human corneal epithelial cells. Mol Vis. 2012;18:758-64.

42. Park JH, Kang SS, Kim JY, Tchah H. Nerve Growth Factor Attenuates Apoptosis and Inflammation in the Diabetic Cornea. Invest Ophthalmol Vis Sci. 2016;57(15):6767-75.

43. Di G, Zhao X, Qi X, Zhang S, Feng L, Shi W, et al. VEGF-B promotes recovery of corneal innervations and trophic functions in diabetic mice. Sci Rep. 2017;7:40582.

44. Wilson SE, Torricelli AAM, Marino GK. Corneal epithelial basement membrane: Structure, function and regeneration. Experimental eye research. 2020;194:108002.

45. Yang L, Di G, Qi X, Qu M, Wang Y, Duan H, et al. Substance P promotes diabetic corneal epithelial wound healing through molecular mechanisms mediated via the neurokinin-1 receptor. Diabetes. 2014;63(12):4262-74.

46. Suvas S. Role of Substance P Neuropeptide in Inflammation, Wound Healing, and Tissue Homeostasis. Journal of immunology (Baltimore, Md : 1950). 2017;199(5):1543-52.

47. Yang L, Sui W, Li Y, Qi X, Wang Y, Zhou Q, et al. Substance P Inhibits Hyperosmotic Stress-Induced Apoptosis in Corneal Epithelial Cells through the Mechanism of Akt Activation and Reactive Oxygen Species Scavenging via the Neurokinin-1 Receptor. PLoS One. 2016;11(2):e0149865.

48. Yanai R, Nishida T, Hatano M, Uchi SH, Yamada N, Kimura K. Role of the Neurokinin-1 Receptor in the Promotion of Corneal Epithelial Wound Healing by the Peptides FGLM-NH2 and SSSR in Neurotrophic Keratopathy. Invest Ophthalmol Vis Sci. 2020;61(8):29.

49. Wang P, Me R, Yuan Y, Yu Y, Li M, Ke B. Substance P inhibits high urea-induced apoptosis through the AKT/GSK-3β pathway in human corneal epithelial cells. J Cell Biochem. 2019.

50. Zhang J, Li H, Wang J, Dong Z, Mian S, Yu FS. Role of EGFR transactivation in preventing apoptosis in Pseudomonas aeruginosa-infected human corneal epithelial cells. Invest Ophthalmol Vis Sci. 2004;45(8):2569-76.

51. Yamada N, Yanai R, Nakamura M, Inui M, Nishida T. Role of the C domain of IGFs in synergistic promotion, with a substance P-derived peptide, of rabbit corneal epithelial wound healing. Invest Ophthalmol Vis Sci. 2004;45(4):1125-31.

52. Yamada N, Yanai R, Inui M, Nishida T. Sensitizing effect of substance P on corneal epithelial migration induced by IGF-1, fibronectin, or interleukin-6. Invest Ophthalmol Vis Sci. 2005;46(3):833-9.

53. Huo Y, Chen W, Zheng X, Zhao J, Zhang Q, Hou Y, et al. The protective effect of EGF-activated ROS in human corneal epithelial cells by inducing mitochondrial autophagy via activation TRPM2. J Cell Physiol. 2020;235(10):7018-29.

54. Miao Q, Xu Y, Zhang H, Xu P, Ye J. Cigarette smoke induces ROS mediated autophagy impairment in human corneal epithelial cells. Environ Pollut. 2019;245:389-97.

55. Huo YN, Chen W, Zheng XX. ROS, MAPK/ERK and PKC play distinct roles in EGF-stimulated human corneal cell proliferation and migration. Cellular and molecular biology (Noisy-le-Grand, France). 2015;61(7):6-11.

56. Zhang J, Wang X, Vikash V, Ye Q, Wu D, Liu Y, et al. ROS and ROS-Mediated Cellular Signaling. Oxid Med Cell Longev. 2016;2016:4350965.

57. Ahmad A, Ahsan H. Biomarkers of inflammation and oxidative stress in ophthalmic disorders. Journal of immunoassay & immunochemistry. 2020;41(3):257-71.

58. Niu L, Li L, Xing C, Luo B, Hu C, Song M, et al. Airborne particulate matter (PM(2.5)) triggers cornea inflammation and pyroptosis via NLRP3 activation. Ecotoxicol Environ Saf. 2021;207:111306.

59. Zhang J, Dai Y, Wei C, Zhao X, Zhou Q, Xie L. DNase I improves corneal epithelial and nerve regeneration in diabetic mice. Journal of cellular and molecular medicine. 2020;24(8):4547-56.

60. Xu KP, Li Y, Ljubimov AV, Yu FS. High glucose suppresses epidermal growth factor receptor/phosphatidylinositol 3-kinase/Akt signaling pathway and attenuates corneal epithelial wound healing. Diabetes. 2009;58(5):1077-85.

61. Li H, Fan TJ, Zou P, Xu B. Diclofenac Sodium Triggers p53-Dependent Apoptosis in Human Corneal Epithelial Cells via ROS-Mediated Crosstalk. Chem Res Toxicol. 2021;34(1):70-9.

62. Mihara M, Erster S, Zaika A, Petrenko O, Chittenden T, Pancoska P, et al. p53 has a direct apoptogenic role at the mitochondria. Molecular cell. 2003;11(3):577-90.

63. Shan M, Fan TJ. Cytotoxicity of carteolol to human corneal epithelial cells by inducing apoptosis via triggering the Bcl-2 family protein-mediated mitochondrial pro-apoptotic pathway. Toxicol In Vitro. 2016;35:36-42.

64. Huo Y, Qiu WY, Pan Q, Yao YF, Xing K, Lou MF. Reactive oxygen species (ROS) are essential mediators in epidermal growth factor (EGF)-stimulated corneal epithelial cell proliferation, adhesion, migration, and wound healing. Experimental eye research. 2009;89(6):876-86.

65. Simon HU, Haj-Yehia A, Levi-Schaffer F. Role of reactive oxygen species (ROS) in apoptosis induction. Apoptosis. 2000;5(5):415-8.

66. Zhang J, Liu Z, Rasschaert J, Blero D, Deneubourg L, Schurmans S, et al. SHIP2 controls PtdIns(3,4,5)P(3) levels and PKB activity in response to oxidative stress. Cellular signalling. 2007;19(10):2194-200.

67. Li J, Qi X, Wang X, Li W, Li Y, Zhou Q. PTEN Inhibition Facilitates Diabetic Corneal Epithelial Regeneration by Reactivating Akt Signaling Pathway. Translational vision science & technology. 2020;9(3):5.

68. Bae YS, Kang SW, Seo MS, Baines IC, Tekle E, Chock PB, et al. Epidermal growth factor (EGF)-induced generation of hydrogen peroxide. Role in EGF receptor-mediated tyrosine phosphorylation. J Biol Chem. 1997;272(1):217-21.

69. Chen KC, Zhou Y, Xing K, Krysan K, Lou MF. Platelet derived growth factor (PDGF)-induced reactive oxygen species in the lens epithelial cells: the redox signaling. Experimental eye research. 2004;78(6):1057-67.

70. Chen KC, Zhou Y, Zhang W, Lou MF. Control of PDGF-induced reactive oxygen species (ROS) generation and signal transduction in human lens epithelial cells. Mol Vis. 2007;13:374-87.

71. Kabza M, Karolak JA, Rydzanicz M, Szcześniak MW, Nowak DM, Ginter-Matuszewska B, et al. Collagen synthesis disruption and downregulation of core elements of TGF-β, Hippo, and Wnt pathways in keratoconus corneas. European journal of human genetics : EJHG. 2017;25(5):582-90.

72. Khaled ML, Bykhovskaya Y, Yablonski SER, Li H, Drewry MD, Aboobakar IF, et al. Differential Expression of Coding and Long Noncoding RNAs in Keratoconus-Affected Corneas. Invest Ophthalmol Vis Sci. 2018;59(7):2717-28.

73. Liu Z, Chen D, Chen X, Bian F, Qin W, Gao N, et al. Trehalose Induces Autophagy Against Inflammation by Activating TFEB Signaling Pathway in Human Corneal Epithelial Cells Exposed to Hyperosmotic Stress. Invest Ophthalmol Vis Sci. 2020;61(10):26.

74. Lu J, Sheng M, Yao P, Ran C, Liu H, Chen L, et al. High Concentration of Glucose Increases Reactive Oxygen Species Generation and Apoptosis Induced by Endoplasmic Reticulum Stress Pathway in Rabbit Corneal Epithelial Cells. J Ophthalmol. 2018;2018:8234906.

75. Wei S, Fan J, Zhang X, Jiang Y, Zeng S, Pan X, et al. Sirt1 attenuates diabetic keratopathy by regulating the endoplasmic reticulum stress pathway. Life Sci. 2021;265:118789.

76. Wang Y, Zhao X, Shi D, Chen P, Yu Y, Yang L, et al. Overexpression of SIRT1 promotes high glucose-attenuated corneal epithelial wound healing via p53 regulation of the IGFBP3/IGF-1R/AKT pathway. Invest Ophthalmol Vis Sci. 2013;54(5):3806-14.

77. Li Y, Li J, Zhao C, Yang L, Qi X, Wang X, et al. Hyperglycemia-reduced NAD(+) biosynthesis impairs corneal epithelial wound healing in diabetic mice. Metabolism: clinical and experimental. 2021;114:154402.

78. Korfi F, Javid H, Assaran Darban R, Hashemy SI. The Effect of SP/NK1R on the Expression and Activity of Catalase and Superoxide Dismutase in Glioblastoma Cancer Cells. Biochem Res Int. 2021;2021:6620708.

79. Rosso M, Muñoz M, Berger M. The role of neurokinin-1 receptor in the microenvironment of inflammation and cancer. ScientificWorldJournal. 2012;2012:381434.

80. Qin L, Wang Z, Tao L, Wang Y. ER stress negatively regulates AKT/TSC/mTOR pathway to enhance autophagy. Autophagy. 2010;6(2):239-47.

81. Jun AS, Meng H, Ramanan N, Matthaei M, Chakravarti S, Bonshek R, et al. An alpha 2 collagen VIII transgenic knock-in mouse model of Fuchs endothelial corneal dystrophy shows early endothelial cell unfolded protein response and apoptosis. Hum Mol Genet. 2012;21(2):384-93.

82. Pierre N, Barbé C, Gilson H, Deldicque L, Raymackers JM, Francaux M. Activation of ER stress by hydrogen peroxide in C2C12 myotubes. Biochem Biophys Res Commun. 2014;450(1):459-63.

83. Park SJ, Kim Y, Yang SM, Henderson MJ, Yang W, Lindahl M, et al. Discovery of endoplasmic reticulum calcium stabilizers to rescue ER-stressed podocytes in nephrotic syndrome. Proc Natl Acad Sci U S A. 2019;116(28):14154-63.

84. Yan Y, Rato C, Rohland L, Preissler S, Ron D. MANF antagonizes nucleotide exchange by the endoplasmic reticulum chaperone BiP. Nat Commun. 2019;10(1):541.

85. Wang X, Li W, Zhou Q, Li J, Wang X, Zhang J, et al. MANF Promotes Diabetic Corneal Epithelial Wound Healing and Nerve Regeneration by Attenuating Hyperglycemia-Induced Endoplasmic Reticulum Stress. Diabetes. 2020;69(6):1264-78.

86. Saghizadeh M, Kramerov AA, Yu FS, Castro MG, Ljubimov AV. Normalization of wound healing and diabetic markers in organ cultured human diabetic corneas by adenoviral delivery of c-Met gene. Invest Ophthalmol Vis Sci. 2010;51(4):1970-80.

87. Spix JK, Chay EY, Block ER, Klarlund JK. Hepatocyte growth factor induces epithelial cell motility through transactivation of the epidermal growth factor receptor. Exp Cell Res. 2007;313(15):3319-25.

88. Xu KP, Yu FS. Cross talk between c-Met and epidermal growth factor receptor during retinal pigment epithelial wound healing. Invest Ophthalmol Vis Sci. 2007;48(5):2242-8.

89. Saghizadeh M, Epifantseva I, Hemmati DM, Ghiam CA, Brunken WJ, Ljubimov AV. Enhanced wound healing, kinase and stem cell marker expression in diabetic organ-cultured human corneas upon MMP-10 and cathepsin F gene silencing. Invest Ophthalmol Vis Sci. 2013;54(13):8172-80.

90. Saghizadeh M, Kramerov AA, Yaghoobzadeh Y, Hu J, Ljubimova JY, Black KL, et al. Adenovirus-driven overexpression of proteinases in organ-cultured normal human corneas leads to diabetic-like changes. Brain research bulletin. 2010;81(2-3):262-72.

91. Edick MJ, Tesfay L, Lamb LE, Knudsen BS, Miranti CK. Inhibition of integrin-mediated crosstalk with epidermal growth factor receptor/Erk or Src signaling pathways in autophagic prostate epithelial cells induces caspase-independent death. Mol Biol Cell. 2007;18(7):2481-90.

92. Cheng CY, Hsieh HL, Hsiao LD, Yang CM. PI3-K/Akt/JNK/NF-κB is essential for MMP-9 expression and outgrowth in human limbal epithelial cells on intact amniotic membrane. Stem cell research. 2012;9(1):9-23.

93. Tokumaru S, Sayama K, Shirakata Y, Komatsuzawa H, Ouhara K, Hanakawa Y, et al. Induction of keratinocyte migration via transactivation of the epidermal growth factor receptor by the antimicrobial peptide LL-37. Journal of immunology (Baltimore, Md : 1950). 2005;175(7):4662-8.

94. Yin J, Yu F-SX. LL-37 via EGFR Transactivation to Promote High Glucose–Attenuated Epithelial Wound Healing in Organ-Cultured Corneas. Investigative Ophthalmology & Visual Science. 2010;51(4):1891-7.

95. Baudouin C, Aragona P, Messmer EM, Tomlinson A, Calonge M, Boboridis KG, et al. Role of hyperosmolarity in the pathogenesis and management of dry eye disease: proceedings of the OCEAN group meeting. The ocular surface. 2013;11(4):246-58.

96. Li H, Li J, Hou C, Li J, Peng H, Wang Q. The effect of astaxanthin on inflammation in hyperosmolarity of experimental dry eye model in vitro and in vivo. Exp Eye Res. 2020;197:108113.

97. Erlandsson Harris H, Andersson U. Mini-review: The nuclear protein HMGB1 as a proinflammatory mediator. Eur J Immunol. 2004;34(6):1503-12.

98. Hwang SB, Park JH, Kang SS, Kang DH, Lee JH, Oh SJ, et al. Protective Effects of Cyclosporine A Emulsion Versus Cyclosporine A Cationic Emulsion Against Desiccation Stress in Human Corneal Epithelial Cells. Cornea. 2020;39(4):508-13.

99. Daull P, Feraille L, Barabino S, Cimbolini N, Antonelli S, Mauro V, et al. Efficacy of a new topical cationic emulsion of cyclosporine A on dry eye clinical signs in an experimental mouse model of dry eye. Experimental eye research. 2016;153:159-64.

100. Daull P, Barabino S, Feraille L, Kessal K, Docquier M, Parsadaniantz SM, et al. Modulation of Inflammation-Related Genes in the Cornea of a Mouse Model of Dry Eye upon Treatment with Cyclosporine Eye Drops. Current eye research. 2019;44(5):476-85.

101. Liu Z, Chen D, Chen X, Bian F, Gao N, Li J, et al. Autophagy Activation Protects Ocular Surface from Inflammation in a Dry Eye Model In Vitro. Int J Mol Sci. 2020;21(23).

102. Elliott EI, Sutterwala FS. Initiation and perpetuation of NLRP3 inflammasome activation and assembly. Immunol Rev. 2015;265(1):35-52.

103. Zhong Z, Umemura A, Sanchez-Lopez E, Liang S, Shalapour S, Wong J, et al. NF-κB Restricts Inflammasome Activation via Elimination of Damaged Mitochondria. Cell. 2016;164(5):896-910.

104. Dakhil TAB, Stone DU, Gritz DC. Adjunctive Therapies for Bacterial Keratitis. Middle East Afr J Ophthalmol. 2017;24(1):11-7.

105. Huang H, Li H, Chen X, Yang Y, Li X, Li W, et al. HMGA2, a driver of inflammation, is associated with hypermethylation in acute liver injury. Toxicol Appl Pharmacol. 2017;328:34-45.

106. Hao C, Zhou Z, Peng C, Gen WX, Ge Z. Inhibitory Effect of Extracellular Polysaccharide EPS-II from Pseudoalteromonas on Candida adhesion to Cornea in vitro. Biomedical and Environmental Sciences. 2012;25(2):210-5.

107. Kernacki KA, Barrett RP, Hobden JA, Hazlett LD. Macrophage inflammatory protein-2 is a mediator of polymorphonuclear neutrophil influx in ocular bacterial infection. Journal of immunology (Baltimore, Md : 1950). 2000;164(2):1037-45.

108. Jaishankar D, Yakoub AM, Yadavalli T, Agelidis A, Thakkar N, Hadigal S, et al. An off-target effect of BX795 blocks herpes simplex virus type 1 infection of the eye. Sci Transl Med. 2018;10(428).

109. Suryawanshi RK, Koujah L, Patil CD, Ames JM, Agelidis A, Yadavalli T, et al. Bacterial Pigment Prodigiosin Demonstrates a Unique Antiherpesvirus Activity That Is Mediated through Inhibition of Prosurvival Signal Transducers. J Virol. 2020;94(13).

110. Qin X, Jiang B, Zhang Y. 4E-BP1, a multifactor regulated multifunctional protein. Cell Cycle. 2016;15(6):781-6.

111. Yadavalli T, Suryawanshi R, Ali M, Iqbal A, Koganti R, Ames J, et al. Prior inhibition of AKT phosphorylation by BX795 can define a safer strategy to prevent herpes simplex virus-1 infection of the eye. The ocular surface. 2020;18(2):221-30.

112. Goswami DG, Tewari-Singh N, Dhar D, Kumar D, Agarwal C, Ammar DA, et al. Nitrogen mustard-induced corneal injury involves DNA damage and pathways related to inflammation, epithelial-stromal separation and neovascularization. Cornea. 2016;35(2):257-66.

113. Xu K-P, Yin J, Yu F-SX. Lysophosphatidic Acid Promoting Corneal Epithelial Wound Healing by Transactivation of Epidermal Growth Factor Receptor. Investigative Ophthalmology & Visual Science. 2007;48(2):636-43.

114. Zhang Z, Liu Z, Meier KE. Lysophosphatidic acid as a mediator for proinflammatory agonists in a human corneal epithelial cell line. American Journal of Physiology-Cell Physiology. 2006;291(5):C1089-C98.

115. Lin CF, Chen CL, Chiang CW, Jan MS, Huang WC, Lin YS. GSK-3beta acts downstream of PP2A and the PI 3-kinase-Akt pathway, and upstream of caspase-2 in ceramide-induced mitochondrial apoptosis. J Cell Sci. 2007;120(Pt 16):2935-43.

116. Xu K, Yu FS. Impaired epithelial wound healing and EGFR signaling pathways in the corneas of diabetic rats. Invest Ophthalmol Vis Sci. 2011;52(6):3301-8.

117. Blobel CP. ADAMs: key components in EGFR signalling and development. Nature reviews Molecular cell biology. 2005;6(1):32-43.

118. Yin J, Yu FS. ERK1/2 mediate wounding- and G-protein-coupled receptor ligands-induced EGFR activation via regulating ADAM17 and HB-EGF shedding. Invest Ophthalmol Vis Sci. 2009;50(1):132-9.

119. Taylor SR, Markesbery MG, Harding PA. Heparin-binding epidermal growth factor-like growth factor (HB-EGF) and proteolytic processing by a disintegrin and metalloproteinases (ADAM): a regulator of several pathways. Semin Cell Dev Biol. 2014;28:22-30.

120. Xu KP, Yin J, Yu FS. SRC-family tyrosine kinases in wound- and ligand-induced epidermal growth factor receptor activation in human corneal epithelial cells. Invest Ophthalmol Vis Sci. 2006;47(7):2832-9.

121. Luttrell DK, Luttrell LM. Not so strange bedfellows: G-protein-coupled receptors and Src family kinases. Oncogene. 2004;23(48):7969-78.

122. Li W, Wang X, Cheng J, Li J, Wang Q, Zhou Q, et al. Leucine-rich α-2-glycoprotein-1 promotes diabetic corneal epithelial wound healing and nerve regeneration via regulation of matrix metalloproteinases. Exp Eye Res. 2020;196:108060.

123. Cheng CC, Wang DY, Kao MH, Chen JK. The growth-promoting effect of KGF on limbal epithelial cells is mediated by upregulation of DeltaNp63alpha through the p38 pathway. J Cell Sci. 2009;122(Pt 24):4473-80.

124. Maeng YS, Lee GH, Lee B, Choi SI, Kim TI, Kim EK. Role of TGFBIp in Wound Healing and Mucin Expression in Corneal Epithelial Cells. Yonsei Med J. 2017;58(2):423-31.

125. Yanai R, Yamada N, Inui M, Nishida T. Correlation of proliferative and anti-apoptotic effects of HGF, insulin, IGF-1, IGF-2, and EGF in SV40-transformed human corneal epithelial cells. Experimental eye research. 2006;83(1):76-83.

126. Tackett BC, Sun H, Mei Y, Maynard JP, Cheruvu S, Mani A, et al. P2Y2 purinergic receptor activation is essential for efficient hepatocyte proliferation in response to partial hepatectomy. Am J Physiol Gastrointest Liver Physiol. 2014;307(11):G1073-87.

127. Wen L, Zhang X, Zhang J, Chen S, Ma Y, Hu J, et al. Paxillin knockdown suppresses metastasis and epithelial‑mesenchymal transition in colorectal cancer via the ERK signalling pathway. Oncol Rep. 2020;44(3):1105-15.

128. Wu YC, Buckner BR, Zhu M, Cavanagh HD, Robertson DM. Elevated IGFBP3 levels in diabetic tears: a negative regulator of IGF-1 signaling in the corneal epithelium. The ocular surface. 2012;10(2):100-7.

129. Shanley LJ, McCaig CD, Forrester JV, Zhao M. Insulin, not leptin, promotes in vitro cell migration to heal monolayer wounds in human corneal epithelium. Invest Ophthalmol Vis Sci. 2004;45(4):1088-94.

130. Nam SM, Maeng YS. Wound Healing and Mucin Gene Expression of Human Corneal Epithelial Cells Treated with Deproteinized Extract of Calf Blood. Curr Eye Res. 2019;44(11):1181-8.

131. Kaplan N, Fatima A, Peng H, Bryar PJ, Lavker RM, Getsios S. EphA2/Ephrin-A1 signaling complexes restrict corneal epithelial cell migration. Invest Ophthalmol Vis Sci. 2012;53(2):936-45.

**Figure Legends**

**Fig. 1 The anatomy of the eye, cornea and corneal epithelium.** As the visual organ of the human body, the eye is a complex and sophisticated structure that consists of the cornea, lens, vitreous, choroid, retina, optic nerve, and other accessory structures from front to back. The cornea is situated at the front of the eye and is composed of the CE, Bowman's layer, corneal stroma, Descemet's membrane and the endothelium from outside to inside. Located at the outermost layer of the cornea, the CE is derived from the surface ectoderm. The CE is a self-renewing stratified nonkeratinized squamous epithelium that protects the inner eye tissue, forms the immune barrier, supports the tear film, and maintains transparency to play a role in transmitting light. CECs include superficial squamous cells, central suprabasal cells, and a single layer of inner columnar basal cells.

**Fig. 2 The PI3K/AKT signalling pathway in CECs.** Damage to CECs, WIN, and CAP can increase the Ca^2+^ concentration in the cytosol, respectively, which induce ectodomain shedding of EGF (and/or HB-EGF) via activation of ADAM and/or MMPs in the plasma membrane and extracellular matrix. The growth factors, insulin, and NK-1 induce PI3K phosphorylation by binding to their receptors in the plasma membrane. Activated PI3K subsequently phosphorylates lipids on the cell membrane to form the second messenger PIP3, leading to the phosphorylation of AKT. Then, AKT mediates downstream responses by phosphorylating or inducing the formation of complexes composed of various downstream molecules, such as mTOR, ROS, and GSK3β. Specifically, mTOR is an important molecule downstream of AKT, and AKT activation indirectly activates mTORC1, promoting cell migration and proliferation, and inhibiting inflammation and apoptosis. AKT, an antiapoptotic factor, also inhibits cell apoptosis through eliminating excessive ROS in cytosol. However, produced by NADPH oxidase, a low or moderate level of ROS act as a second messenger of PI3K and inhibitor of Phosphatase and tensin homologue, both of which promote activation of AKT. Activated AKT also phosphorylates GSK3β and reduces its activity, thereby promoting cell migration and proliferation. All of the above effects promote the healing of the CE together. NOX, NADPH oxidase; INSR, Insulin receptor; IGFBP3, Insulin-like growth factor-binding protein 3; α3β1-INT, α3β1 integrin; IRS2, Insulin receptor substrate 2; TSC2, Tuberous sclerosis complex 2; Bax, Bcl-2-associated X protein; Bad, Bcl-2-associated agonist of cell death protein; Bim, Bcl-2-like protein 11; CytC, cytochrome c; PXN, Paxillin; PCNA, Proliferating cell nuclear antigen; CDK, Cyclin-dependent kinase; PTEN, Phosphatase and tensin homologue; WIN, MAP kinase kinase kinase win; CAP, capsaicin; CB1, cannabinoid receptor 1; TRPV1, transient receptor potential vanilloid 1; PARP, poly (ADP-ribose) polymerase; MIP-1β, macrophage inflammatory protein 1β; MIP-1α, macrophage inflammatory protein 1α; ADAM, a disintegrin and metalloproteinase.

**Fig. 3 The roles of different molecules related to the PI3K/AKT pathway in CECs in diseases and therapeutic strategies.** The PI3K/AKT signalling pathway plays an important role in DK, DED and keratitis. Many exogenous or endogenous molecules affect the development of corneal diseases through the PI3K/AKT signalling pathway. Furthermore, many newly discovered drugs alleviate these corneal diseases by regulating the PI3K/AKT signalling pathway. This figure uses different colours to indicate different corneal diseases and clarify the complex signalling network in CECs. In DK, hyperglycaemia induces insulin production, promotes the activation of the PI3K/AKT signalling pathway, and inhibits cell apoptosis. On the other hand, hyperglycaemia increases ROS levels. It inhibits IGF1 receptor, EGFR-ErbB2, TGF-β, GPCR, and other AKT upstream molecules, inducing cell apoptosis and inhibiting corneal wound healing. DED-induced hyperosmolarity inhibits the PI3K/AKT pathway and activates cell apoptosis and autophagy, leading to corneal inflammation. CsA and AST were also discovered reverse the changes caused by DED through the PI3K/AKT pathway. However, in noninfectious and infectious keratitis, overactivated AKT promotes inflammation. LPS, NM, and EPS-II increase the levels of inflammation-related proteins by activating the PI3K/AKT pathway. Moreover, as potential drugs, AA, C3G, and other molecules inhibit inflammation through the PI3K/AKT pathway. In particular, in viral keratitis, the PI3K/AKT pathway is also an important target for regulating viral (HSV-1) replication. GPCR, G protein-coupled receptor; AA, asiatic acid; NM, Nitrogen mustard; NAC, N-acetyl cysteine; MANF, Mesencephalic astrocyte-derived neurotrophic factor; TFEB, Transcription factor; PERK, Protein kinase RNA- like endoplasmic reticulum kinase; CHOP, C/EBP-homologous protein; VEGF, Vascular endothelial growth factor; COX2, Cyclooxygenase 2; CF, cathepsin F; HSV-1, herpes simplex virus type 1.

**Fig. 4 The localization and function of different molecules involved in the PI3K/AKT signalling pathway in the CE.** At the tissue level, different molecules affect CECs by modulating the PI3K/AKT signalling pathway *in vivo* and *in vitro*. Some factors are extracellular, such as wounds, some drugs (AST, CsA, AA, and C3G), and some biological or chemical stimulants (LPS, EPS, and NM). Moreover, some factors exist in cells, such as glucose, insulin, various growth factors related to hyperglycaemia, and hyperosmolarity, which is related to DED.

**Table 1. Classic molecules in PI3K/AKT signalling in CECs.**

| **Molecular name** | **Molecular Function** | **Biological Function in CECs** | **Regulators** | **Subcellular Location** | **Contributors** |
| --- | --- | --- | --- | --- | --- |
| Phosphatase and tensin homologue | Tumor suppressor, inhibit PI3K/AKT signalling | Apoptosis and inhibition of proliferation | / | Cytoplasm, cell cortex | [66] |
| PIP2, PIP3, PI3K | Direct regulators of AKT | Proliferation and anti-apoptosis | Phosphatase and tensin homologue | Cytoplasm, cell cortex | [66] |
| AKT | Upstream regulator of mTORC1, Forkhead box protein O1, and GSK3α/GSK3β | Proliferation and anti-apoptosis | PIP3, PI3K | Cytoplasm, nucleus | [66] |
| Tuberous sclerosis complex 2, mTOR complex 1 (mTORC1) | Substrates of AKT | Growth and anti-apoptosis | AKT | Cytoplasm | [43] |
| Forkhead box protein O1 | Inhibited by AKT | Apoptosis and inhibition of proliferation | AKT | Cytoplasm and nucleus | [66] |
| Cyclic AMP-responsive element-binding protein 1 | Transcription of Bcl-2 | Proliferation and anti-apoptosis | AKT | Nucleus, Golgi apparatus, endoplasmic reticulum membrane | [95] |
| GSK3β | Activated by AKT, inhibiting GYS1/2 to reduce Glycogen synthesis, inhibiting MYC | Apoptosis and anti-proliferation | AKT | Cytoplasm | [49] |
| p70S6K, rpS6 | Substrate of AKT1/mTORC1, related to Glucose homeostasis and protein synthesis | Proliferation and anti-apoptosis | mTORC1 | Cytoplasm | [115] |
| eIF5A, Eukaryotic translation initiation factor 4E-binding protein 1 | Target of p70S6K | Proliferation and anti-apoptosis | p70S6K, rpS6 | Nucleus and cytosol | [25] |
| Cyclin A1/2  Cyclin D1/3  Cyclin E1/3 | Regulating the G1/S/G2/M transitions | Proliferation | S-phase kinase-associated protein 2, Myc proto-oncogene protein | Nucleus | [41] |
| Proliferating cell nuclear antigen | DNA damage response, DNA repair | Proliferation and anti-apoptosis | eIF5A | Nucleus | [116] |
| Bcl-2 | Activated by Cyclic AMP-responsive element-binding protein 1, inhibiting apoptosis | Proliferation and anti-apoptosis | Cyclic AMP-responsive element-binding protein 1 | Cytoplasm | [63] |
| Bcl-2-associated X protein, Bcl-2-like protein 11, Bcl-2-associated agonist of cell death protein, Cytochrome c | Apoptosis regulator in mitochondria | Mitochondria-induced apoptosis | ROS | Mitochondrion | [63, 98] |
| Cas-3, 8, 9 | Apoptosis caused by DNA damage and cleaving poly (ADP-ribose) polymerase | Apoptosis | Cytochrome c | Cytoplasm | [115] |
| P53 | Tumor suppressor, stimulating Bax, inhibiting Bcl-2 | Apoptosis | mTORC1 | Nucleus and mitochondrion | [40, 76] |
| A disintegrin and metalloproteinase 9, 10, 12, 17  MMP-3, 8, 9, 10 | Local proteolysis of extracellular matrix (fibronectin, gelatins, collagens), establishing and maintaining gradients | Migration | rpS6, Src, Wound | Extracellular matrix, plasma membrane | [117, 118] |
| IL-1β, IL-1α | Potent proinflammatory cytokine | Inflammation | LPS, ROS, TNF-α | Lysosome, cytosol | [48] |
| TNF-α | Pathogen defense, inflammation | Inflammation and apoptosis | / | Plasma membrane | [96] |

**Table 2. The function of different molecules related to PI3K/AKT pathway in CECs and CECs-related diseases.**

| **Molecular name** | **Molecular Function** | **Biological function in CECs** | **Regulators** | **Subcellular location** | **Contributors** |
| --- | --- | --- | --- | --- | --- |
| HB-EGF | EGFR and ErbB2 binding | Wound healing, cell migration, proliferation | Src; MMP 3,7；a disintegrin and metalloproteinase 9, 10, 12, 17; | Plasma membrane | [116, 119] |
| EGF, EGFR | Activating PI3K/AKT signalling | Anti-apoptosis | HB-EGF | Plasma membrane | [119] |
| Poly (ADP-ribose) polymerase | DNA repair | Anti-apoptosis | Cas-3, 8, 9 | Nucleus | [120] |
| ErbB2 | Receptor of HB-EGF | Anti-apoptosis | HB-EGF | Plasma membrane and nucleus | [121] |
| Src | Inducing ectodomain shedding of HB-EGF, and playing as second messenger of EGFR | Proliferation and anti-apoptosis | Ca^2+^, EGFR, ErbB2 | Cytoplasm | [120] |
| TGF-β | Activate PI3K/AKT and MAPK signalling pathway | Epithelial mesenchymal transition, keratitis, wound healing | Peucine-rich alpha-2-glycoprotein | Extracellular region | [122] |
| MAPK(P38) | Inhibtied by EGF signalling and inhibiting TGF-β | Apoptosis | EGF | Cytosol and nucleus | [123] |
| Transforming Growth Factor β-Induced Protein | Induced by TGF-β, binding with α3β1 integrin | Adhesion | TGF-β | Extracellular matrix | [124] |
| eIF5A, PCNA | The substrate of EGF/EGFR/Src/PI3K/AKT signalling pathway | Cell migration and proliferation | EGF | Nucleus | [25] |
| MMP-9 | The substrate of EGF/EGFR/Src/PI3K/AKT signalling pathway | Cell migration and proliferation | EGF, NM | Cytosol and extracellular matrix | [25] |
| LPA | Transactivate EGFR and activate Src/PI3K/AKT signalling pathway | Wound healing, inflammation | Protein phosphatase 2 | Extracellular region | [113, 114] |
| G protein coupled receptor | transactivation of AKT signalling | Proliferation and anti-apoptosis | LPA | Plasma membrane | [125] |
| P2Y purinoceptors | Multi-pass membrane protein for influx of extracellular Ca^2+^ | Proliferation and anti-apoptosis | ATPγS | Plasma membrane | [126] |
| LL-37 | Bind to LPS and transactivate EGFR | Proliferation, anti-apoptosis and anti-bacterial | Wound | Plasma membrane | [126] |
| Capsaicin, cannabinoid receptor 1 | Mobilization from intracellular Ca^2+^ stores | Proliferation and anti-apoptosis | / | Plasma membrane and nucleus | [33] |
| Mitogen-activated protein kinase kinase kinase win, transient receptor potential vanilloid 1 | Influx of extracellular Ca^2+^ | Proliferation and anti-apoptosis | / | Plasma membrane and nucleus | [33] |
| Paxillin | Cytoskeletal protein associated with actin-membrane attachment, correlating with integrin | Adhesion(anti-migration) | GSK3β | Cell cortex and cytoskeleton | [127] |
| Focal adhesion kinase | Activation of PI3K/AKT, maintaining focal adhesion, binding with cytosol part of integrin | Adhesion(anti-migration), proliferation, anti-apoptosis | Paxillin, Integrin | Nucleus, plasma membrane, cytoskeleton | [127] |
| IGF1, IGF1R | Activating PI3K/AKT signalling | Cell adhesion, proliferation, survival | Insulin, IGF binding protein 3 | Nucleus, plasma membrane, cytoplasm | [35, 76] |
| IGFBP3 | Binding with IGF1 to inhibit IGF1/IGF1R | Apoptosis, adhesion | P53 | Secreted | [128] |
| Leucine-rich alpha-2-glycoprotein 1 | Activating TGFβR, promoting the expression of MMP-3 and MMP-13 | Wound healing | Glucose | Secreted | [122] |
| SIRT1 | Enhancing IGFBP3/IGF-1R/AKT pathway | Proliferation and anti-apoptosis | NK-1R, p53 | Nucleus, cytoplasm, mitochondrion | [76] |
| Insulin, INSR | Activating PI3K/AKT signalling, blocks GSK3β activity | Wound healing, cell migration, mitophagy and mitochondrial accumulation | Glucose | Extracellular region | [39, 129] |
| HGF, Keratinocyte growth factor | Activating PI3K/AKT/p70S6K signalling pathway | Wound healing and cell proliferation | IL-1, IL-6, TNFα, glucose | Extracellular region | [40] |
| Nerve growth factor | Activating PI3K/AKT signalling pathway, reduce the expression of Caspase-3,9, Bad, Bax and Bim | Cell growth and G1-S transition | / | Endosome and extracellular region | [41] |
| Vascular endothelial growth factor-B, Vascular endothelial growth factor receptor-1 | Promote the expression of pigment epithelium-derived factor via PI3K/AKT/GSK3β/mTOR signalling | Wound healing and regeneration of nerve fiber | Vascular endothelial growth factor trap | Extracellular region | [43] |
| NK-1, NK-1R | Reactivate EGFR, AKT, and SIRT1 signalling | Wound healing, corneal sensation recovery and mitochondrial function recovery | NK-1 receptor antagonist | Extracellular region and plasma membrane | [45] |
| ROS | Suppressor of EGFR-mediated PI3K/AKT signalling pathway, increase the expression of Bcl-2, Bax, cytochrome c, Caspase-3, 8, and 9 | Delay wound healing, delay regeneration and migration, apoptosis, Inflammation, keratoconus | N-acetylcystein | Cytoplasm and mitochondrion | [53-55, 61] |
| MAPK3/1 | MAPK3/1 signalling pathway synergistics with PI3K/AKT signalling pathway | Anti-apoptosis, wound healing, cell survival | Dual specificity mitogen-activated protein kinase kinase mek | Cytoplasm and cytoskeleton | [130] |
| NAD^+^ | Increasing ROS, inhibit  SIRT1/EGFR/PI3K/AKT signalling pathway | Mitochondria-induced apoptosis,  delay wound healing in diabetes | Glucose | Cytoplasm and mitochondrion | [77] |
| Nicotinamide phosphoribosyltransferase | Increasing ROS, inhibit  SIRT1/EGFR/PI3K/AKT signalling pathway | Mitochondria-induced apoptosis,  delay wound healing in diabetes | Glucose | Cytoplasm, nucleus, extracellular region | [77] |
| Mesencephalic astrocyte-derived neurotrophic factor | Activate AKT signalling | Inhibits hyperglycaemia-induced ER stress and ER stress-mediated  apoptosis, wound healing, nerve regeneration in diabetes | Glucose | Endoplasmic reticulum and extracellular region | [85] |
| MMP-10, Cathepsin F | Downstream of EGFR/PI3K/AKT signalling pathway | Delay wound healing in diabetes | Glucose | Extracellular region and lysosome | [89] |
| Ephrin-A1, A2 | Suppressing AKT signalling | Attenuates cell migration, delay wound healing in diabetes | Glucose | Plasma membrane and extracellular region | [131] |
| HMGB1 | Related to PI3K/AKT signalling | Immune response, tissue damage in DED | AST | Plasma membrane, nucleus, endosome cytoplasm, extracellular region | [96] |
| CsA | Inhibit the expression of TNF-α, Bax and Bcl-2 via reactivating PI3K/AKT signalling pathway | Reduce apoptosis and inflammation | / | Extracellular region and cytoplasm | [98] |

1. [↑](#footnote-ref-1)
