# Supplementary material for: The role of the PI3K/AKT signalling pathway in the corneal epithelium: recent updates
Source: Cell Death Dis. 2022 May 31;13(5):513. doi: 10.1038/s41419-022-04963-x (PMC9156734; doi:10.1038/s41419-022-04963-x)
Supplement: Supplementary file 2 — Title, running title and abstract-R3 [file 41419_2022_4963_MOESM2_ESM.docx]

**The role of the PI3K/AKT signalling pathway in the corneal epithelium: Recent updates**

Kuangqi Chen^^[[1]](#footnote-1)^^, Yanqing Li^1^, Xuhong Zhang^1^, Rahim Ullah^2^, Jianping Tong^1^ and Ye Shen^1^

Correspondence: Ye Shen ([idrshen@zju.edu.cn](mailto:idrshen@zju.edu.cn))

Jianping Tong (idrtong@zju.edu.cn)

^1^ Department of Ophthalmology, the First Affiliated Hospital, School of Medicine, Zhejiang University, Hangzhou, zhejiang, 310003, PR China; Kuangqi Chen ([3160105142@zju.edu.cn](mailto:3160105142@zju.edu.cn)); Yanqing Li (3180103182@zju.edu.cn); Xuhong Zhang (11918408@zju.edu.cn);

^2^ Department of Endocrinology, Children's Hospital of Zhejiang University School of Medicine, National Clinical Research Center for Child Health, Hangzhou, Zhejiang, 310052, PR China; Rahim Ullah ([Rahim@zju.edu.cn](mailto:Rahim@zju.edu.cn));

*These authors contributed equally: Kuangqi Chen, Yanqing Li

**Running title：The role of PI3K/AKT signalling pathway in the corneal epithelium.**

**Abstract:** Phosphatidylinositol 3 kinase (PI3K)/AKT (also called protein kinase B, PKB) signalling regulates various cellular processes, such as apoptosis, cell proliferation, the cell cycle, protein synthesis, glucose metabolism, and telomere activity. Corneal epithelial cells (CECs) are the outermost cells of the cornea; they maintain good optical performance and act as a physical and immune barrier. Various growth factors, including epidermal growth factor receptor (EGFR) ligands, insulin-like growth factor 1 (IGF1), neurokinin 1 (NK-1), and insulin activate the PI3K/AKT signalling pathway by binding their receptors and promote antiapoptotic, anti-inflammatory, proliferative, and migratory functions and wound healing in the corneal epithelium (CE). Reactive oxygen species (ROS) regulate apoptosis and inflammation in CECs in a concentration-dependent manner. Extreme environments induce excess ROS accumulation, inhibit PI3K/AKT, and cause apoptosis and inflammation in CECs. However, at low or moderate levels, ROS activate PI3K/AKT signalling, inhibiting apoptosis and stimulating proliferation of healthy CECs. Diabetes-associated hyperglycaemia directly inhibit PI3K/AKT signalling by increasing ROS and endoplasmic reticulum (ER) stress levels or suppressing the expression of growth factors receptors and cause diabetic keratopathy (DK) in CECs. Similarly, hyperosmolarity and ROS accumulation suppress PI3K/AKT signalling in dry eye disease (DED). However, significant overactivation of the PI3K/AKT signalling pathway, which mediates inflammation in CECs, is observed in both infectious and noninfectious keratitis. Overall, upon activation by growth factors and NK-1, PI3K/AKT signalling promotes the proliferation, migration, and anti-apoptosis of CECs, and these processes can be regulated by ROS in a concentration-dependent manner. Moreover, PI3K/ AKT signalling pathway is inhibited in CECs from individuals with DK and DED, but is overactivated by keratitis.

**Keywords:** PI3K, AKT, corneal epithelial cells, corneal epithelium, wound healing

**Facts**

CECs are present in the outermost layer of the cornea and play an important role in maintaining CE homeostasis and optical function.

The PI3K/AKT signalling pathway is widely involved in the proliferation, apoptosis, migration, and other functions of CECs.

Many corneal diseases and wound healing are associated with the PI3K/AKT signalling and its interaction with ROS.

**Open questions**

How do CECs respond to different environmental conditions, such as health and disease states, through the PI3K/AKT signalling pathway?

How does the PI3K/AKT signalling pathway interact with ROS in CECs to control the cellular response and influence disease progression?

Can the PI3K/AKT signalling pathway be used as a major target for the treatment of corneal diseases in the future?

1. [↑](#footnote-ref-1)
