# Supplementary material for: The role of the PI3K/AKT signalling pathway in the corneal epithelium: recent updates
Source: Cell Death Dis. 2022 May 31;13(5):513. doi: 10.1038/s41419-022-04963-x (PMC9156734; doi:10.1038/s41419-022-04963-x)
Supplement: Supplementary file 4 — Editing Certificate of AJE [file 41419_2022_4963_MOESM4_ESM.pdf]

Editing Certificate

This document certifies that the manuscript

The role of the PI3K/AKT signalling pathway in the corneal epithelium: Recent updates.

prepared by the authors

Kuangqi Chen, Yanqing Li, Xuhong Zhang, Rahim Ullah, Jianping Tong and Ye Shen

was edited for proper English language, grammar, punctuation, spelling, and overall style by one or more of the highly qualified native English speaking editors at AJE.

This certificate was issued on **April 1, 2022** and may be verified on the AJE website using the verification code **5D88-E04E-2BAF-F508-DAEP** .

Neither the research content nor the authors' intentions were altered in any way during the editing process. Documents receiving this certification should be English-ready for publication; however, the author has the ability to accept or reject our suggestions and changes. To verify the final AJE edited version, please visit our verification page at aje.com/certificate. If you have any questions or concerns about this edited document, please contact AJE at support@aje.com.

AJE provides a range of editing, translation, and manuscript services for researchers and publishers around the world. For more information about our company, services, and partner discounts, please visit aje.com.
